# Enhanced data quality to improve malaria surveillance in Papua, Indonesia

**DOI:** 10.1186/s12936-025-05358-x

**Published:** 2025-06-04

**Authors:** Liony Fransisca, Faustina Helena Burdam, Enny Kenangalem, Annisa Rahmalia, Reynold Rizal Ubra, Christel H. A. van den Boogaard, Benedikt Ley, Nicholas M. Douglas, Jeanne Rini Poespoprodjo, Ric N. Price

**Affiliations:** 1 Yayasan Pengembangan Kesehatan Dan Masyarakat Papua (YPKMP), Timika, Indonesia; 2 Mimika Regency Health Office, Timika, Indonesia; 3 Mimika General Hospital, Timika, Indonesia; 4 Institute of Tropical Medicine, Antwerp, Belgium; 5 Menzies School of Health Research, Charles Darwin University, Darwin, Australia; 6 Department of Medicine, University of Otago, Christchurch, New Zealand; 7 Department of Infectious Diseases, Christchurch Hospital, Te Whatu Ora, Christchurch, New Zealand; 8 Centre for Child Health-pro, Faculty of Medicine, Public Health and Nursing Universitas Gadjah Mada, Yogyakarta, Indonesia; 9 Nuffield Department of Clinical Medicine, Centre for Tropical Medicine and Global Health, University of Oxford, Oxford, UK; 10 Mahidol-Oxford Tropical Medicine Research Unit (MORU), Faculty of Tropical Medicine, Mahidol University, Bangkok, Thailand

**Keywords:** Malaria, Surveillance, Continuous quality improvement, CQI, Data quality, Prescribing accuracy

## Abstract

**Background:**

Papua has a high burden of malaria, with an annual parasite incidence 300 times the national average. A key component of malaria elimination strategies is robust surveillance which is essential for monitoring trends in case numbers, guiding public health interventions, and prioritizing resource allocation. This study aimed to enhance malaria surveillance in Central Papua, Indonesia, by improving data collection, record-keeping, and treatment practices.

**Methods:**

The study was conducted at five public clinics in Central Papua province, Indonesia, as part of a wider health systems strengthening programme to promote safer and more effective anti-malarial treatment (The SHEPPI Study). Clinical and laboratory details of patients with malaria and their treatment were documented in clinic registers which were digitalized into an electronic database. Automated reports were generated each month and used to provide regular feedback to clinic staff. Continuous Quality Improvement (CQI) workshops were conducted with clinic staff using the Plan-Do-Study-Act approach to address challenges and drive sustained improvements.

**Results:**

Between January 2019 and December 2023, a total of 314,561 patients were tested for malaria, of whom 41.9% (131,948) had peripheral parasitaemia detected. The first round of Continuous Quality Improvement (CQI) workshops were held in May 2019 and improved data quality significantly, increasing data completeness from 46.3% (4540/9802) in the initial period (Jan–May 2019) to 71.5% (9053/12,665) after the first CQI (Jun–Oct 2019), p < 0.001. The second CQI round reduced DHP prescribing errors from 17.1% (1111/6489) in the initial period to 5.7% (607/10,669) after the second CQI (Sep 2019–Jan 2020) and PQ prescribing errors from 17.4% (552/3175) to 3.4% (160/4659) over the same time interval, p < 001. In total, 347 patients were prescribed fewer than the recommended number of PQ tablets during the initial period, 89 (25.6%) of whom were erroneously given only a single dose. Over the 4 year study period, a total of 11 workshops were conducted, driving continuous improvements in data quality and prescribing practices.

**Conclusion:**

One or two rounds of CQI, supported by regular follow-up, can enhance the quality of malariometric surveillance, however interventions needed to be tailored to address specific needs of participating clinics. Improvements in data quality and prescribing practices have potential to contribute to better malaria management, improved clinical outcomes, and strengthened trust in healthcare providers.

**Supplementary Information:**

The online version contains supplementary material available at 10.1186/s12936-025-05358-x.

## Background

Malaria continues to exert a huge global health burden. In 2023, an estimated 263 million malaria cases and 597,000 deaths were reported worldwide, with the main burden of disease occurring in sub-Saharan Africa. In the last two decades the global malaria incidence and associated mortality have fallen, but more recently, progress towards elimination goals has slowed down and even reversed in some areas [1]. Malaria surveillance plays a crucial role in defining these temporal and geographical trends, supporting public health systems and ensuring the efficient use of resources [2–4].

In Indonesia, approximately 40 million people or 15% of the population live in malaria-endemic areas. The burden of malaria is particularly high in the eastern provinces where *Plasmodium falciparum* and *Plasmodium vivax* infections are equally prevalent [5]. The incidence of malaria tends be greatest in remote rural areas, although significant numbers of cases are also reported from urban areas associated with higher population densities [6, 7]. The true incidence of malaria in Indonesia is unknown since in poorly resourced areas, laboratory-confirmation is rare and less than 40% of patients with symptomatic malaria seek treatment at government healthcare facilities [8].

Mimika Regency, located in Central Papua province, has the highest burden of malaria in Indonesia, with an annual parasite incidence greater than 400 per 1000 population in 2023, almost 300 times higher than the national average [9–11]. Developed and implemented by the Ministry of Health, the Indonesian national electronic malaria surveillance system (eSISMAL) is used widely by public government hospitals and clinics, to facilitate standardized malaria case reporting across the country and to provide data on patient demographics and laboratory results [12, 13]. Relevant data for these reports are gathered from the outpatient department, laboratory, and pharmacy records. At clinics with internet access, malaria programme officers entered the data directly into an online application, whereas where internet access was unavailable, a paper record was prepared and uploaded to the online application once a connection was available. This process is time consuming and usually incomplete, particularly in remote and high-endemic areas, hence eSISMAL does not provide comprehensive data across all endemic areas. Furthermore, the data gathered do not provide insights into prescribing accuracy or recurrence rates.

Recurrent episodes of malaria can be due to parasite recrudescence (treatment failure due to inadequate treatment or anti-malarial drug resistance), reinfection from a new mosquito bite, or, in the case of *P. vivax*, relapses from reactivation of dormant hypnozoites, weeks to months after an initial infection [14–17]. Whilst it is not possible to distinguish the causes of recurrent malaria without advanced molecular analysis, high rates of recurrence highlight the need for further investigation and broader malaria control strategies to reduce disease burden and decrease parasite transmission [18]. Quantifying the risk of recurrent infection requires identifying patients through recurrent encounters with the healthcare system [19]. Although national identification cards can facilitate tracking patients through multiple encounters, patients frequently forget to bring their identification cards with them, and thus healthcare providers may be unable to link medical records for the same patient. Surveillance and case management is also undermined by healthcare staff who are burdened by high workloads and hindered by incomplete or inaccurate demographic and clinical data. The latter can impair quality improvement processes and lead to under-allocation of resources. Reporting errors are further compounded by delays in collating the data and reporting, and this prevents timely decision-making and resource allocation [20, 21].

In 2019, in consultation with the local government, an enhanced malariometric surveillance system was established at five government-run public health facilities in the Mimika district of Central Papua, Indonesia. The project was titled ‘Strengthening health systems for effective primaquine in Papua Indonesia’ (SHEPPI). The aim of the SHEPPI study was to facilitate data collection, provide regular reports on data quality and clinic workloads, evaluate the local malaria epidemiology and treatment practices, and quantify the proportion of patients presenting with recurrent malaria due to any species.

This paper, reports the impact of enhanced malaria surveillance on the data quality of clinic records and anti-malarial prescribing accuracy at five clinics. It also summarizes the epidemiology and temporal trends of patients presenting with any species of malaria over a four-year period, to demonstrate the utility of more robust surveillance systems.

## Methods

This study was observational in nature seeking to document normal clinical practice with minimal direct intervention from research staff at the interface between the patient and healthcare provider. However, the study also included an interventional aspect provided by the continuous quality improvement meetings.

## Site details

Five study clinics (Timika, Timika Jaya, Wania, Pasar Sentral, and Bhintuka) were selected to represent varying patient burdens and a mixture of urban and rural settings, to provide an overview of the healthcare dynamics in the region (Appendix 1). Mimika spans 21,522 square kilometres and consists of 12 sub-districts with a total of 85 villages. Fragmented forests extend from coastal lowlands to mountainous regions. Mimika receives an average of 5.5 m of rainfall annually, with peak rainy seasons occurring between July and September and again in December. Significant economic migration, driven by the presence of a local mine, has contributed to a diverse population comprising highland Papuans, lowland Papuans, and non-Papuans. Most of the population resides in lowland zones below 1600 meter in altitude, where malaria transmission occurs consistently throughout the year, with little seasonal variation. The primary mosquito vectors responsible for malaria transmission are *Anopheles koliensis*, *Anopheles farauti*, and *Anopheles punctulatus*. These mosquitoes are opportunistic in their host-seeking behaviours and exhibit both exophilic and endophilic tendencies [22].

## Clinical management of patients with malaria

At all five study clinics, patients presenting with fever were screened for malaria by microscopic examination of thick and thin blood films. The parasite species was assessed by trained microscopists from Giemsa-stained thick blood films and peripheral parasitaemia determined from the number of parasites per 200 white blood cells. A thick smear was considered negative on initial review if no parasites were seen in 100 high power fields. In three clinics, a thin smear was also examined to confirm parasite species and used for quantification if parasitaemia was greater than 200 per 200 WBC. When blood film examination could not be done due to electricity outage or after service hours, patients were tested with a malaria rapid diagnostic test (RDT). A variety of RDTs were used by the clinics including First Response (Premier Medical Corporation, India), Plasmotec (Plasmotec Pte Ltd, Singapore), CareStart (Access Bio, USA), and Standard Q Malaria Pf/Pan Ag RDT (SD Biosensor, South Korea).

Patients diagnosed with malaria were treated according to national malaria treatment guidelines. The first-line schizontocidal treatment for patients with uncomplicated malaria due to any species was dihydroartemisinin plus piperaquine (DHP). Patients with *P. falciparum* malaria were treated with a single dose of primaquine (PQ) 0.25 mg/kg for gametocytocidal activity. Patients over the age of 6 months with *P. vivax* or *Plasmodium ovale* malaria were treated with a 14 day regimen of primaquine 0.25 mg/kg/day, unless they were pregnant or breast feeding. The national guidelines recommend that patients representing after 28 days with recurrent *P. vivax* malaria, are presumed to have had a relapse and can be treated with a higher dose of primaquine (0.5 mg/kg/day for 14), providing patients are confirmed to have normal G6PD activity. During the study period, G6PD testing was not available at any of the primary health care facilities in Mimika. Patients with severe malaria were referred to the local hospital for medical management.

## Data collection and record-keeping

At each of the study clinics, a study nurse worked with local clinic staff to improve record-keeping practices and subsequently monitor data quality. Clinic staff recorded routine patient and laboratory data in the clinic register, including that required for eSISMAL reports. As part of the SHEPPI study, additional data were also recorded on the patients’ weight and details of the number of anti-malarial tablets prescribed (Appendix 2). This detailed system of monitoring, focusing on collecting comprehensive data to improve malaria control and prevention efforts is referred to as enhanced malariometric surveillance. All relevant malaria data were collated at a dedicated desk (called the ‘malaria corner’), where patients with malaria were provided with education on malaria, medical treatment, and first dose supervision. The malaria corner at each clinic was run by local clinic staff supported by the study nurse. A ‘malaria card’ system was introduced in a staggered fashion from August 2019 to February 2020. Each patient with malaria was provided with a card containing a unique identification number to present to the clinic staff in the event the patient had to return to the clinic with fever or other symptoms consistent with malaria.

## Data entry and management

From October 2019, the study nurse was tasked with entering clinical and laboratory data for malaria patients from the clinic register into an electronic database separate from eSISMAL, which includes data from 1 st January 2019. Data were initially collected into an EpiData database (EpiData Association, Denmark), but in December 2022, data entry transitioned to a web-based REDCap database (Vanderbilt University, Nashville, TN) hosted by Menzies School of Health Research for enhanced security. Both database systems ensured standardized data collection across participating clinics. Database management was carried out in collaboration with the District Health Office to ensure compliance with national data protection laws and regulations. At the start of the SHEPPI project, the focus of data assessment was on data integrity (completeness, accuracy, consistency) and the quality of clinical management, with solutions formulated in subsequent CQI workshops.

Healthcare providers’ adherence to the national malaria treatment guidelines was categorized according to the the number of tablets prescribed and the patients’ age and/or weight (Appendix 3). Instances where patients were prescribed fewer or more tablets than recommended were identified and categorized according to whether the administered dose was too high, too low, or aligned with the dosing for an adjacent weight band. The total primaquine dose prescribed was compared to the target total of 3.5 mg/kg.

## Automated reporting and feedback mechanisms

Digitalized data were used to generate automated monthly reports, presenting a summary of clinic workloads and associated demographics of patients as well as metrics on data quality and completeness. Data were considered missing if no value was entered or if the value was recorded as unknown. Legality parameters were set in both epidata and REDCap to maximize data fidelity at the point of entry and further standardized data screening algorithms were developed in STATA (College Station, Texas, USA) prior to data analysis.

Accuracy of anti-malarial prescribing was evaluated by assessing concordance between the weight/age-based drug doses prescribed and the doses recommended in Indonesian national malaria treatment guidelines (Appendix 3). Automated reports were shared periodically with clinic staff to provide feedback on any issues with data integrity or case management (Appendix 4).

## Continuous quality improvement (CQI)

Continuous Quality Improvement (CQI) workshops were held periodically every 3–6 months to provide feedback and foster ongoing improvements in both clinical practice and data accuracy (Appendix 5). These workshops were attended by the heads of health centres, malaria corner staff, malaria programme officers at the health centre and regency level, and the head of disease prevention and control section in the Regency Health Office. CQI applied the Plan-Do-Study-Act (PDSA) approach to assist the District Health Officers and staff at the five study clinics identify problems, set achievable goals, implement plans, and reassess improvements. This approach systematically tests small changes on a limited scale so that learning and adaptation can be done prior to scaling up the whole change. Despite the impact of the COVID19 pandemic, 11 CQI workshops were convened between May 2019 and September 2023.

## Statistical analysis

The statistical analyses were conducted according to an a priori statistical plan (Appendix 6), using STATA statistical software (Version 18, Stata Corp, Texas, USA). The Mann–Whitney U test or Kruskal–Wallis method was used for nonparametric comparisons, and Student’s t-test or one-way analysis of variance for parametric comparisons. For categorical variables, percentages and corresponding 95% confidence intervals (95% CI) were calculated using Wilson’s method. Proportions were examined using Chi squared tests with Yates'correction or by Fisher's exact test. Since the first CQI meeting was held 5 months after the start of the surveillance system, time period bins of 5 months were chosen for comparison of outcomes during the study period. Time periods for comparing outcomes were categorized into an the initial period (January to May 2019), 5 months after the 1 st CQI (June to October 2019), 5 months after the 2nd CQI (September 2019 to January 2020), and the final study period (August to December 2023).

## Results

### Malaria epidemiology in Timika

Between January 2019 and December 2023, a total of 314,561 patients were tested for malaria across all five study clinics: 299,801 (95.3%) by microscopic blood film examination and 14,760 (4.7%) by RDT. The positivity rate was 41.2% (123,396/299,801) by microscopy and 57.9% (8552/14,760) by RDT (Table S1). The proportion of cases due to *P. vivax* remained consistent throughout the study (ranging from 42.1 to 47.7%), but there was a rise in the proportion of cases due to *Plasmodium malariae*, from 2.2% (589/26,856) in 2019 to 7.5% (2269/30,153) in 2023 (p = 0066; Fig. S1, Table S2). Approximately 0.36% (467/131,927) of patients with malaria were identified as having recurrent parasitaemia within 30 days due to mono or mixed *P. falciparum* infection and 0.37% (485/131,927) were due to recurrence with mono or mixed *P. vivax* infections.

**Fig. 1:**
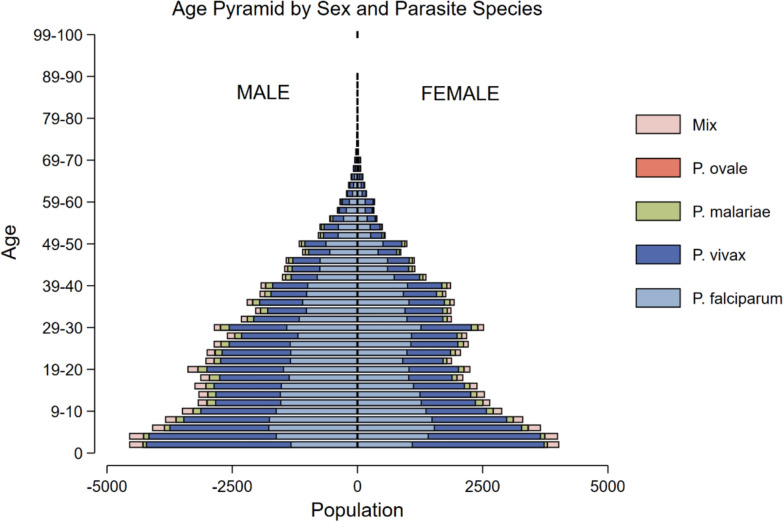
Age sex distribution for malaria patients, 2019–2023

The age distribution of patients with malaria differed significantly between the species of infection, p < 0.001. Overall, 40.4% (52,973/130,951) of patients with malaria were children (< 15 years old). Among adults, *P. vivax* accounted for 40.4% (31,393/77,789) of cases, while *P. falciparum* accounted for 49.4% (38,413/77,789). In children aged 5–15 years, *P. vivax* contributed to 42.7% (13,594/31,860) of cases compared to 47.1% (15,000/31,860) for *P. falciparum*. Among children under 5 years old, *P. vivax* caused 58.0% (12,240/21,113) of cases, while *P. falciparum* accounted for 33.8% (7128/21,113). Males made up 55.1% (72,135/130,951) of all malaria cases, and this proportion remained consistent across species and over time (Fig. 1, Table 1, Table S2).

**Table 1:** Baseline Characteristic by species, 2019–2023

Category	Pf (N = 60,665)	Pv (N = 57,284)	Pm (N = 6109)	Po (N = 267)	Mix (N = 6626)	Total (N = 130,951)
Age groups						
< 1 y.o	359 (0.6%)	1016 (1.8%)	16 (0.3%)	1 (0.4%)	66 (1.0%)	1458 (1.1%)
1–5 y.o	6769 (11.2%)	11,224 (19.6%)	411 (6.7%)	31 (11.6%)	1220 (18.4%)	19,655 (15.0%)
5–15 y.o	15,000 (24.7%)	13,594 (23.7%)	1,439 (23.6%)	66 (24.7%)	1761 (26.6%)	31,860 (24.3%)
> 15 y.o	38,413 (63.3%)	31,393 (54.8%)	4237 (69.4%)	168 (62.9%)	3578 (54.0%)	77,789 (59.4%)
Unknown	124 (0.2%)	57 (0.1%)	6 (0.1%)	1 (0.4%)	1 (0.0%)	189 (0.1%)
Weight based on age groups, mean kg (SD)						
< 1 y.o	7.8 (3.3)	7.6 (2.4)	6.3 (1.7)	9.0 (0)	8.8 (11.1)	7.7 (3.5)
1–5 y.o	13.9 (5.2)	13.0 (4.2)	14.3 (5.5)	13.7 (2.3)	13.5 (3.5)	13.4 (4.6)
5–15 y.o	32.8 (13.3)	32.1 (13.4)	33.2 (12.7)	33.5 (13.8)	33.0 (13.7)	32.5 (13.3)
> 15 y.o	61.0 (12.4)	60.5 (12.6)	60.4 (12.0)	61.6 (12.5)	59.7 (12.0)	60.7 (12.5)
Unknown	49.9 (16.8)	43.4 (24.8)	44.0 (29.5)	0 (0.0%)	57.3 (0)	48.0 (19.7)
Sex						
Male	33,171 (54.7%)	31,569 (55.1%)	3517 (57.6%)	151 (56.6%)	3727 (56.2%)	72,135 (55.1%)
Female	27,436 (45.2%)	25,629 (44.7%)	2590 (42.4%)	116 (43.4%)	2,895 (43.7%)	58,666 (44.8%)
Unknown	58 (0.1%)	86 (0.2%)	2 (0.0%)	0 (0.0%)	4 (0.1%)	150 (0.1%)
Pregnancy status						
Not pregnant	25,959 (94.6%)	24,385 (95.1%)	2495 (96.3%)	115 (99.1%)	2802 (96.8%)	55,756 (95.0%)
Pregnant	1128 (4.1%)	1032 (4.0%)	55 (2.1%)	1 (0.9%)	84 (2.9%)	2300 (3.9%)
Unknown	349 (1.3%)	212 (0.8%)	40 (1.5%)	0 (0.0%)	9 (0.3%)	610 (1.0%)
Lactating status						
Not lactating	27,300 (99.5%)	25,460 (99.3%)	2587 (99.9%)	116 (100.0%)	2880 (99.5%)	58,343 (99.4%)
Lactating	136 (0.5%)	169 (0.7%)	3 (0.1%)	0 (0.0%)	15 (0.5%)	323 (0.6%)
Current presentation is a recurrence within 30 days						
Any	402 (0.66%)	450 (0.78%)	16 (0.26%)	1 (0.37%)	67 (1.01%)	936 (0.71%)
Mono/mix Pf	280 (0.46%)	142 (0.25%)	9 (0.15%)	0 (0%)	36 (0.54%)	467 (0.36%)
Mono/mix Pv*	135 (0.22%)	308 (0.54%)	2 (0.03%)	1 (0.37%)	39 (0.59%)	485 (0.37%)

Data are N (%), unless otherwise indicated. Data were derived from 130,951 (99.2%) of cases in which species of infection was recorded

^*^Includes Pv/Pan result from RDT

### Temporal changes in malaria during the study period

**Fig. 2:**
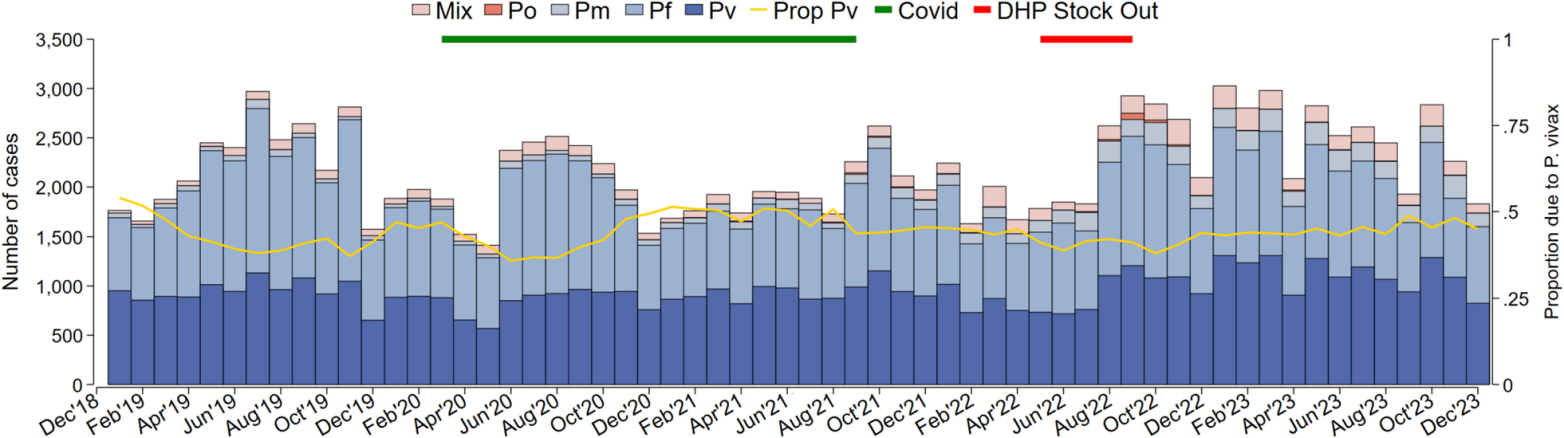
Malaria cases per month by Plasmodium species for all clinics, 2019–2023

The number of malaria cases declined at the start of the COVID- 19 pandemic (Fig. 2). This was reflected in a significant decrease in the number of malaria tests conducted during the pandemic (78,053 tests in 2019, 51,639 in 2020, 53,648 in 2021, 58,817 in 2022) and subsequent rise after the end of the pandemic (72,404 in 2023).

Between May and September 2022, a national shortage of DHP resulted in a stockout of DHP at all clinics, though primaquine remained available.These patients were treated with 7 days of quinine. In the first three months of the stockout, (May to July 2022) there was no change in the mean number of malaria cases (1819 cases per month), however the number of cases rose by 53.8% to an mean of 2797 cases per month over the following three months (August to October 2022) beyond the end of the stock out. During this post stock out period, the proportion of malaria due to each species did not change.

### Quality of data and clinical management

In the initial period (January to May 2019), a total of 9,802 patients with malaria presented to the five clinics. There was a high proportion of missing or erroneously recorded data particularly for medical record numbers (52.5%, 5118/9802), weight (29.5%, 2892/9802), and pregnancy status in females (10.9%, 482/4430). In contrast, minimal missing data were observed for sex (1.4%, 133/9802) and age (1.1%, 107/9,802).

After the first CQI (June to October 2019), the proportion of missing data or data errors fell to 25.7% 3252/12,665 for medical record numbers, 5.5% (694/12,665) for weight and 1.8% (99/5514) for pregnancy status, p < 0.001. The data quality improved further after the second CQI (September 2019 to January 2020) and was sustained until the final period of the study report (Table S3). Overall, in the initial period of the study, 46.3% (4540/9802) of reported malaria cases had complete records for medical record number, sex, age, weight, parasite data, and this rose to 78.5% (8706/11,087) after the second CQI and to 91.1% (10,290/11,301) in the final period of 2023, p < 0.001 (Table S4).

In addition, the malaria card system was introduced in Wania clinic in August 2019, Pasar Sentral and Bhintuka clinics in September 2019, Timika Jaya clinic in October 2019, and lastly in Timika clinic in February 2020. In the final period of the study, 62.9% (7104/11,301) of patients had malaria card numbers assigned to them during clinic visits.

To assess the accuracy of prescribing practices, data on the number of DHP and PQ tablets prescribed along with age and/or weight need to be recorded. Data availability for schizontocidal treatment (DHP) improved over time, rising from 68.6% (6724/9802) in the initial five months, to 96.0% (12,160/12,665) after the first CQI and 99.4% (11,020/11,087) after the second CQI, p < 0.001 (Table S5). The corresponding availability of data from which to calculate PQ prescription accuracy for patients with *P. vivax* or *P. ovale* infection rose initially from 70.2% (3364/4793) to 96.0% (5261/5477) after the first CQI and 99.2% (4949/4987) after the second CQI workshop, p < 0.001 (Table S6).

**Fig. 3:**
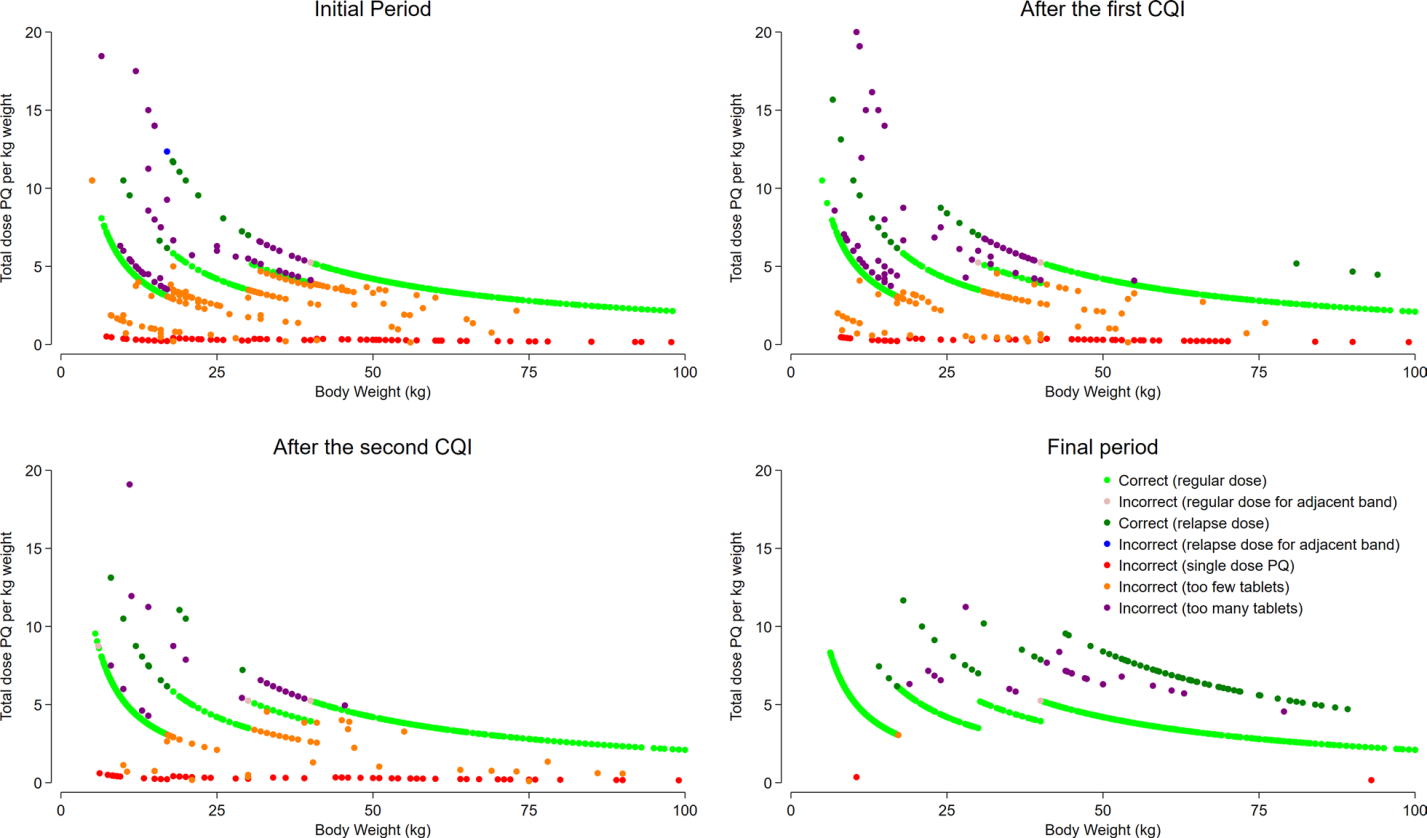
Comparison of the prescribed DHA dose with the recommended dose from national guidelines, based on the number of tablets

**Fig. 4:**
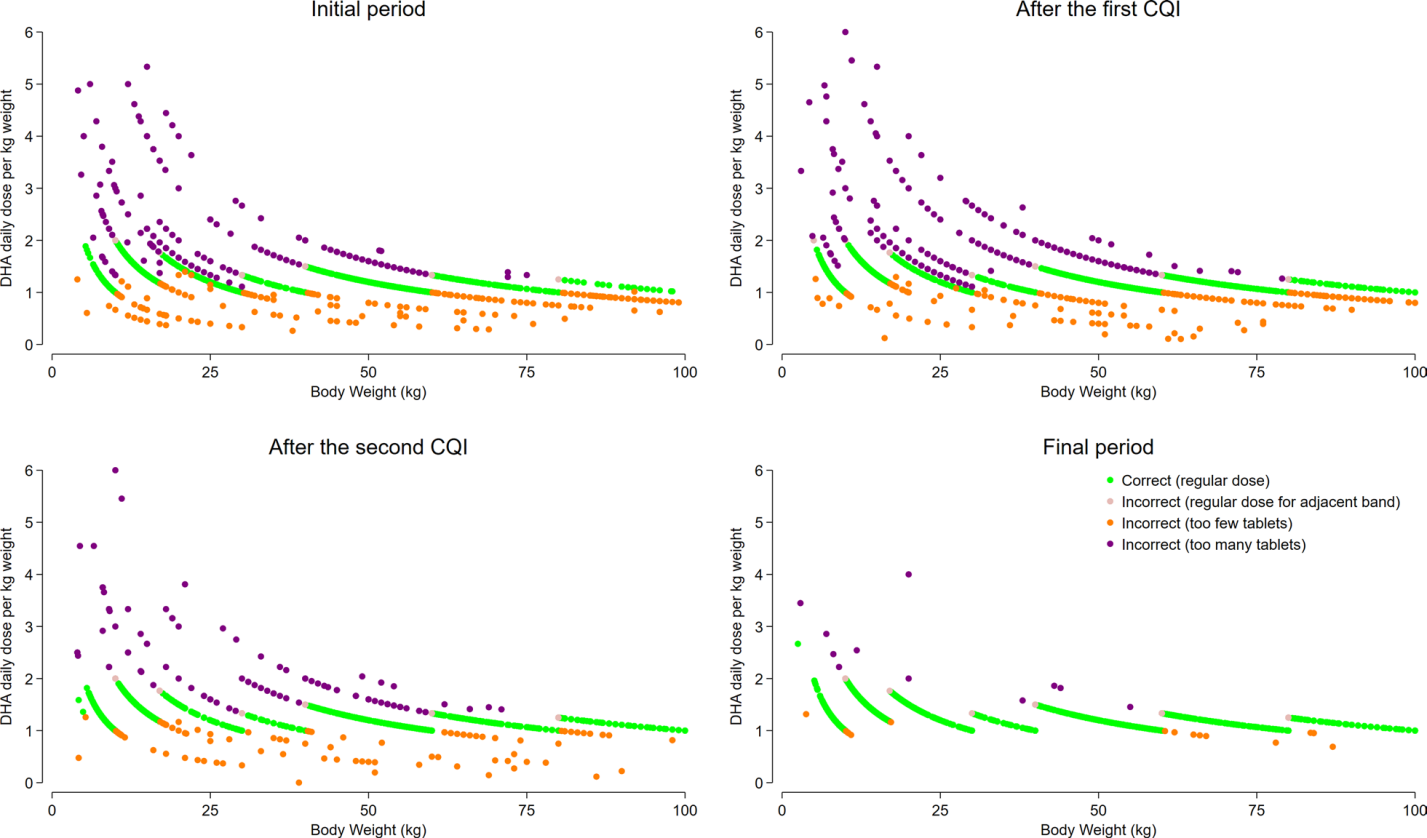
Comparison of the prescribed Piperaquine dose with the recommended dose from national guidelines, based on the number of tablets

In total, 96.5% (6489/6724) of patients with available data were prescribed DHP during the initial 5 months period, of whom 17.1% (1111/6489) were prescribed the incorrect number of tablets (Figs. 3 and 4). Overall, 43.9% (488/1111) were prescribed fewer than the recommended number of tablets, and 56.1% (623/1111) were prescribed more than the recommended number of tablets. Of the latter, 18.1% (113/623) were prescribed tablets for an adjacent weight band due to borderline weight. Most of the errors in prescribing were apparent at two clinics (Fig. S2). The proportion of patients prescribed an incorrect dose of DHP did not differ by sex. However, patients with *P. vivax* malaria had a lower odds of receiving an incorrect dose compared to those with *P. falciparum* (Odds Ratio: 0.79 [95% CI 0.74–0.85], p < 0.001). Additionally, children under 1 year of age were significantly more likely to be prescribed an incorrect dose compared to those 15 years or older (Odds Ratio: 2.50 [95% CI 2.02–3.08], p < 0.001).

During the initial period, 98.7% (3321/3364) of patients with *P. vivax* or *P. ovale* malaria who had available data were eligible to be treated with PQ, of whom 95.6% (3175/3321) were prescribed PQ. A total of 552 (17.4%) of these patients were prescribed the incorrect number of tablets; 62.9% (347/552) of these dosing errors were due to prescription of fewer than the recommended number of tablets, including 25.6% (89/347) erroneously prescribed a single dose of PQ. The remaining 37.1% (205/552) of errors were prescriptions for more than the recommended number of tablets. Overall, 4.3% (137/3175) were prescribed less than a total dose of 2 mg/kg and 0.38% (12/3175) were prescribed an equivalent daily dose of > 0.75 mg/kg/day. The proportion of patients prescribed an incorrect dose of 14 days PQ did not differ by sex nor species. However, children under 1 year of age were significantly more likely to be prescribed an incorrect dose compared to those 15 years or older (Odds Ratio: 2.80 [95% CI 1.79–4.38], *p* < 0.001).

There was significant improvement in prescribing accuracy following the first CQI workshop conducted in May 2019. The proportion of patients prescribed the correct dose of DP rose from 82.9% (5378/6489) in the first 5 months to 91.0% (10,798/11,864) after the first CQI to 93.4% (10,062/10,669) and this was sustained until the end of the (Table S7). The corresponding proportion of patients with *P. vivax* or *P. ovale* prescribed the correct dose of PQ rose from 82.6% (2623/3175) in the initial period to 93.3% (4654/4987) after the first CQI to 96.6% (4499/4659) after the second CQI and 99.5% (5744/5775) at the end of the study (Table S7, S8).

**Fig. 5:**
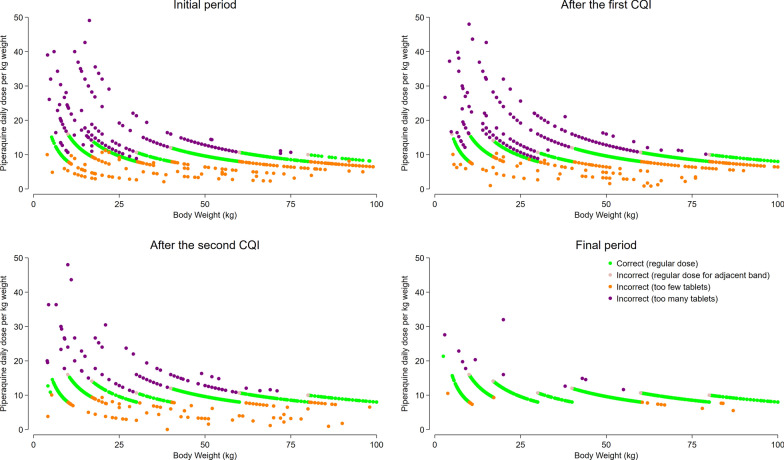
Comparison of the prescribed PQ dose with the recommended dose from national guidelines, based on the number of tablets

Although prescribing accuracy according to the national malaria treatment guidelines improved during the study, the applied weight-stratified dose banding resulted in deviation from the target total primaquine dose of 3.5 mg/kg (Fig. 5). Overall, the median total dose of PQ prescribed to patients with *P. vivax* malaria was 3.8 mg/kg [0.13–18.5] in the first months of the study and 4.0 mg/kg [0.2–9.0] at the end of the study; p < 0.001.

**Fig. 6:**
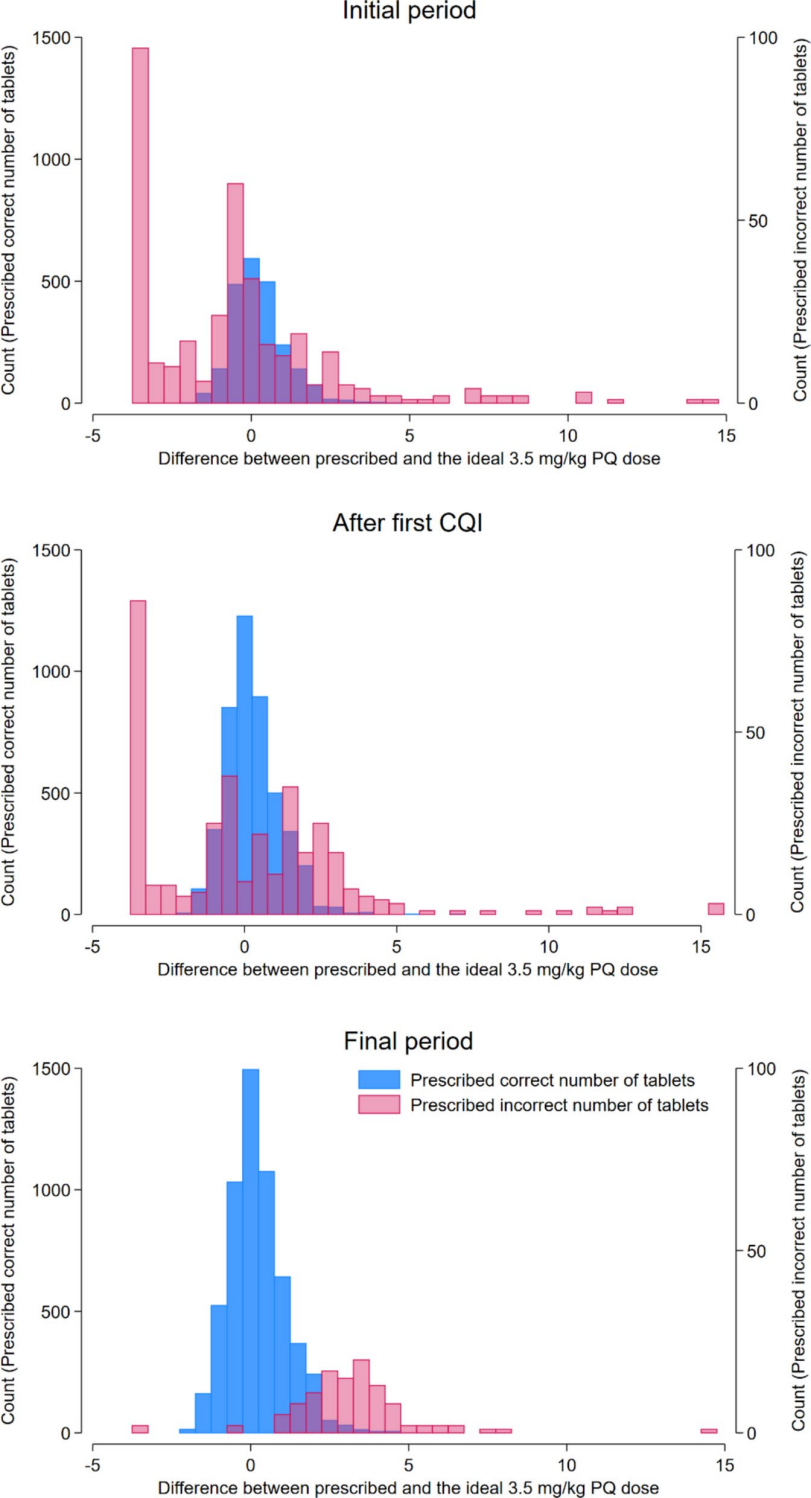
Comparison of the prescribed dose with the target total PQ dose of 3.5 mg/kg

In the initial period, the PQ dose in patients who were prescribed the correct number of tablets according to national guidelines ranged from 2.1 to 8.1 mg/kg. The range of primaquine dose was even greater in patients incorrectly prescribed PQ (0.1 to 18.5 mg/kg). During the final period of the study, the PQ dose for correctly prescribed patients ranged from 2.1 to 8.3 mg/kg, and for incorrectly prescribed patients from 0.2 to 11.7 mg/kg. Although the range of PQ dose differences narrowed as prescribing habits improved, there was still a noticeable variation from the recommended total dose (Fig. 6).

## Discussion

Early diagnosis and prompt treatment are essential for malaria elimination, but the ability to deliver this requires robust surveillance to facilitate mobilization of resources and patient access to treatment [23]. The primary aim of this health strengthening study was to enhance malariometric surveillance by improving the quality of data gathered and support good clinical practice in the five clinics. Significant variability in data quality can be observed (Table S3). In the initial period of the project, more than half of patient records documented in the clinic register were incomplete.

Poor record keeping can arise from inadequate training of healthcare staff, lack of standardized protocols, or staff simply being overburdened by high case numbers. High clinic workloads can also result in patients receiving suboptimal treatment and this excerbates the situation by leading to higher rates of recurrence and case numbers. Identifying patients with recurrent malaria is challenging since relevant identification is frequently unavailable because physical cards may have been misplaced or simply not brought to clinic.

Continuous Quality Improvement (CQI) can play a major role in enhancing professional practices and healthcare outcomes, especially when collaboration and communication among healthcare professionals are central to the activities. Regular, participant-led meetings within multidisciplinary teams, face-to-face training coupled with audit and feedback have been shown to be more effective than web-based alternatives in fostering competence and motivation. CQI is most effective when all four key methodological features are observed: iterative cyclic methods, continuous data collection, small-scale testing, and clear articulation of the theoretical rationale of the projects [24]. Interaction among quality improvement teams fosters normative pressure, capacity building, and peer recognition [25]. Furthermore active involvement of local decision-makers, such as the District Health Office, can enhance mutual learning, and facilitate resource allocation [26].

In this study, a continuous quality improvement (CQI) process was undertaken, using a Plan-Do-Study-Act model. Automated reports were generated from digitalized data and fed back to clinic staff in a series of CQI cycles in which staff training was offered. Whilst this model has been shown to improve clinical processes and patient outcomes, previous studies have used inconsistent reporting and frequent adaptations have made it difficult to assess their effectiveness [27]. In Central Papua, the CQI process was associated with data completeness increasing by greater than 50% after the first CQI cycle, with almost 80% complete records after the second CQI cycle, and > 90% by the end of the study period in 2023 (Table S4).

Improvements in routine data collection, including recording of patients’ weight and the exact number of tablets that were prescribed, allowed assessment of whether patients were being treated according to national guidelines and calculation of the exact mg/kg dose of DHP and primaquine that they were prescribed. After a single CQI training workshop four clinics achieved > 90% data completeness required to assess dosing, and this was sustained throughout the study period. By the final study period, the newer version of eSISMAL included a weight variable, further supporting the efforts to improve data completeness.

The study confirms the ongoing high burden of malaria in Papua, Indonesia. In this area, febrile patients are routinely tested for malaria either by microscopy or RDT. Over the five-year period of this surveillance study, more than 300,000 patients at the 5 study clinics were tested for malaria. The slide positivity rate ranged from 35% in Bhintuka to 44% in Timika; these rates were similar irrespective of whether patients were tested by microscopy or RDT (Table S1). During 2019–2020, *P. falciparum* exhibited a slightly higher positivity rate overall; however, the *P. falciparum* to *P. vivax* ratio became more balanced in the subsequent years.

Young children are at particularly high risk of *P. vivax* malaria, which accounts for almost 60% of malaria in children under the age of 5 years, whereas *P. falciparum* is the dominant cause of malaria in adults (Fig. 1). These findings align with trends in the Asia Pacific region, where *P. falciparum* typically predominates but *P. vivax* continues to represent a significant portion of the malaria burden, particularly in younger populations [28–31].

The rise in the proportion of malaria due *to P. vivax* in many co-endemic locations highlights the challenges in killing the liver stages of the parasite that sustain ongoing transmission [32–34]. The WHO currently recommends a 14-day regimen of primaquine for patients with *P. vivax* malaria, and adherence to such a long course of treatment is often poor, reducing its effectiveness, which has been reported to be as low as 10% [35, 36] Although the low-dose primaquine regimen (total dose 3.5 mg/kg) is the most widely used regimen in vivax endemic countries including Indonesia, a recent pooled analysis highlighted that a higher total dose (7 mg/kg) provides better antirelapse efficacy [37–39]. In areas without G6PD testing this may increase the risk of drug-induced haemolysis. In clinical practice, the effectiveness of low dose primaquine can be further compromised by reluctance of healthcare providers to prescribe the drug, as well as errors in prescribing the correct dose or inadequate dosing adherence by patients or their caregivers. Prescribing safe and effective primaquine treatment is challenging, potentially leading to high rates of recurrent infections, and associated risks of anaemia and impaired growth and development in young children [40–42].

During the onset of the COVID- 19 pandemic in 2020, there was a decline in malaria testing, and this was associated with a reduction in the number of malaria cases reported (Fig. 2), a likely reflection of restriction of community activities, which may have discouraged patients from attending healthcare facilities. The early stage of the COVID- 19 pandemic also saw significant reductions in outpatient visits and malaria testing in other countries [43]. Healthcare provision was also impacted by a national stockout of DHA-piperaquine, the first-line anti-malarial for all species of malaria, which began in May 2022 and lasted 6 months (Fig. 2). The switch to suboptimal regimens including oral quinine and provision of anti-malarials in the private sector, was associated with a 50% increase in malaria cases that extended for 2 months beyond the end of the stock out.

The study sought to provide information on potential means to improve the clinical management of patients with malaria. The enhanced data collected allowed the study team to gauge the accuracy of DHP and PQ prescriptions, which varied significantly between clinics. Initially 30–40% of patients did not receive the nationally recommended treatment in two clinics, although dosing accuracy was > 85% in the three other clinics. In the two clinics where prescribing accuracy was poor, there was an inverse relationship between prescribing accuracy and the number of malaria cases, accuracy declining significantly as case workload increased (Fig. S2). Prescibing accuracy could be improved rapidly with a single round of CQI although two rounds were needed at one clinic to reach acceptable performance. The approach taken as part of these health strengthening activities highlights the importance of Pillar 3 of the WHO malaria strategy (Transforming malaria surveillance into a key intervention) and its potential to deliver better anti-malarial treatments which in turn are critical for improving health outcomes and ultimately achieving the elimination of malaria [2].

The initial automated reports and CQI meetings revealed errors in both case recording and prescription, but it was difficult to determine whether improvements in prescribing habits were attributable to better record keeping or changes in prescribing practice. The first round of CQI focused primarily on improving data quality, and this led to a rapid improvement in treatment records. But improvements in prescribing adherence took longer and became most evident after the second round of CQI, which specifically addressed this issue. However, it is possible that some of the apparent prescription errors could reflect errors in documentation of weight or number of tablets.

The clinical implications of incorrect dosing depend on the magnitude and direction of the error. Underdosing increases the risk of treatment failure, recurrent episodes of malaria and can also facilitate the emergence and spread of drug resistance [44, 45]. Conversely, overdosing can result in a higher likelihood of adverse events [46]. In the first 5 months of the study, a commonly observed error was the incorrect prescription of a single dose of PQ for patients with *P. vivax* malaria (Fig. 5, panel A). Another common cause of dosing errors was receiving a dose intended for an adjacent weight band. Concurrently, many patients received an incorrect number of tablets for reasons that could not be determined. During the first and second CQI cycle, training on prescribing was addressed and this led to a rapid improvement in prescribing accuracy, particularly at the two poorly performing clinics. In the final period of the study, greater than 98% of malaria patients across all clinics received the correct dose of PQ and < 0.05% of patients with *P. vivax or P. ovale* were prescribed a single dose of PQ Fig. 5.

Current national malaria treatment guidelines recommend PQ treatment according to 5 weight bands and this inevitably leads to deviation from the desired target total dose of 3.5 mg/kg (Fig. 6). In patients prescribed the correct number of tablets according to national guidelines the total dose of PQ administered ranged from 1.5 to 5 mg/kg. Hence whilst refresher training of clinic staff helped reduce prescribing errors, slightly revised national guidelines could ensure more precise dosing. Ideal anti-malarial dosing is further undermined by the lack of paediatric anti-malarial formulations, which, until the end of 2023, made it challenging to adjust doses accurately for small children. Lack of paediatric formulations and concerns regarding drug tolerability may result in healthcare staff prescribing insufficient doses of DHP and primaquine to children, particularly in infants less than 1 year old who are vulnerable to recurrent episodes of malaria and associated morbidities. A similar finding was reported in Ghana, where less than one in five prescribers correctly prescribed DHP for managing malaria in very young infants [47].

This healthcare strengthening programme employed a research nurse at each study clinic to promote better recording practices. The study clinics relied on physical medical records, with unique patient identification remaining a challenge. However, in health facilities utilising electronic medical records and incorporating national ID or universal medical insurance cards, fewer human resources may be required for such tasks. In these cases, a single support staff could potentially alternate between different clinics, the incentive being that modest efforts can result in constructive improvements in surveillance and case management that will ultimately impact on clinical outcomes such as the risk of clinical deterioration and recurrent episodes of malaria. Further studies are now underway to explore better ways of tracking individual patients through the healthcare system so that the impact of improved case management on clinical outcomes can be assessed.

The study has several limitations. Data were gathered in an observational manner with minimal direct intervention at the point of care. Hence it is not possible to determine whether improvements in prescribing habits were due to better record-keeping, better delivery of care or a combination of both factors. Furthermore, the health system strengthening activities were only conducted at 5 clinics, although these were selected to be representative of local public health facilities. Whilst the CQI approach was highly effective in improving data quality, it involved a research nurse at each clinic. The study was not designed to address the feasibility, acceptability, sustainability and scalability of the intervention. Studies are now underway to specifically address this as part of a larger implementation of a novel intervention package designed to provide safe and more effective radical cure of *P. vivax* malaria.

## Conclusion

This study demonstrates that data quality required for malariometric surveillance can be improved rapidly through a single round of CQI and that this can be sustained with modest input. However, while the CQI approach is broadly effective, individual clinics may respond differently to the intervention, and the time required to achieve necessary improvements varies between sites. This underscores the importance of adapting training workshops for staff according to available local data, to address clinic-specific challenges and ensure consistent progress in both data quality and clinical management. Data-driven insights can inform and guide targeted interventions, that can improve case management and clinical outcomes of patients with malaria and build community trust in healthcare providers.

## Supplementary Information

Supplementary Material 1.

## Data Availability

The datasets used and/or analysed during the current study are available from the corresponding author on reasonable request.
