# Supplementary material for: Enhanced data quality to improve malaria surveillance in Papua, Indonesia
Source: Malar J. 2025 Jun 4;24:177. doi: 10.1186/s12936-025-05358-x (PMC12135496; doi:10.1186/s12936-025-05358-x)
Supplement: Supplementary file 1 — Supplementary Material 1. [file 12936_2025_5358_MOESM1_ESM.pdf]

APPENDIX

Appendix 1. Map of health facilities in Timika

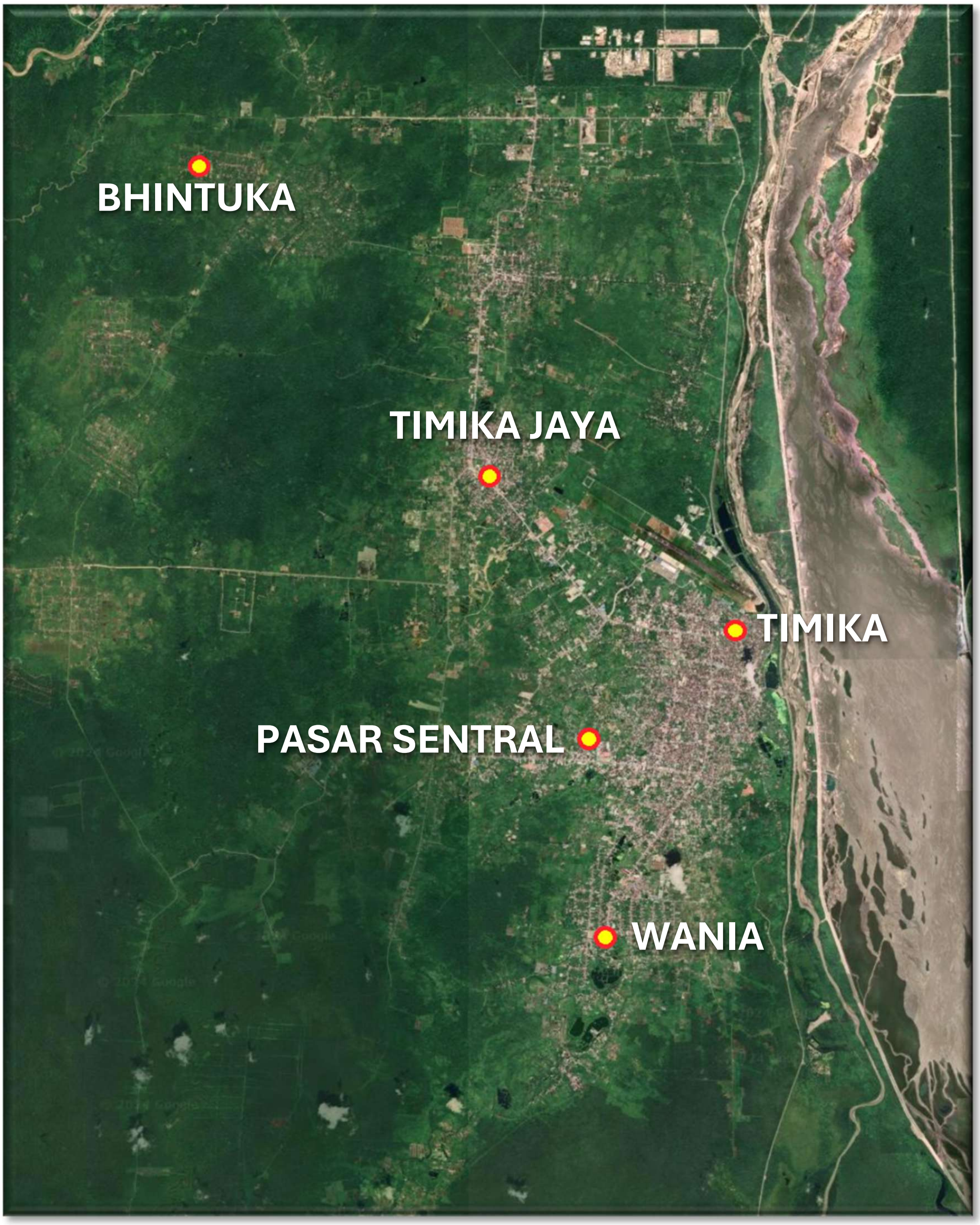

Appendix 2. SHEPPI project data code book

**Variable name-Ind**	**V1**	**V2**	**Variable name-Eng**	**format**	**Comment**	
PUSKESMAS	x	x	Health care centre name	# (name from dropdown list)<br>1 PKM_TIMIKA<br>2 PKM_TIMIKA_JAYA<br>3 PKM_PASAR_SENTRAL<br>4 PKM_WANIA<br>5 "PKM_BHINTUKA (SP13)"	From drop down box, shouldn't be typos	
TANGGAL	x	x	date of visit	<dd/mm/yyyy>	Date limit: <2018 ; >2021	
MONTH	x	x	month of visit	## (range 1-12) dropdown list	automatically filled from date	
CLINMATCH	x	x	are there lab results for this patient?	# (0=no; 1=yes)		
CARA	x	x	?	# (1=passive; 2=active)	Automatically 1 'passive' will be filled out	
Identity			***Identity***			
NUMB	x	x	Number given in this data base, sequential per clinic and time of registration	#####		
REGISTER	x	x	register number, irregular	<AAAAAAAAAAAA>		
NKK	x	x	National ID number, most missing, people don't give this	<AAAAAAAAAAAA>		
Data Pasien			***Patient data***			
NAMAPASIEN	x	x	Patient name	<AAAAAAAAAAAAAAAAAAAA>		
Umur:			*Age:*			
TAHUN	x	x	age in years	### yrs (0-120,999=unknown)	should be filled out for everybody, even if < 1 yrs of age then there should be a 0<br>Warning>80 and >100, please check very old	
BULAN	x	x	age in months	### mnths (0-11, 99=unknown)	should be filled out for <1yr of age	

SEX	x	x	gender	# (1=male; 2=female)		
HAMIL	x	x	pregnant	#(0=no;1=yes;9=unknown)	Male cannot be pregnant<br>Women age <=12; >=50 yrs very unlikely to be pregnant	warning
BERAT	x	x	weight in kg	###.# kg	weight - age bands based on hh survey<br><2yrs 2-15kg<br>>=2, <5yrs 10-30kg<br>>=5, <10yrs 15-40kg<br>>=10, <15yrs 25-60kg<br>>=15yrs 35-90kg<br>(WHO 0-5yrs 2-25 kg; 5-10 12-60kg)	warning
ALAMAT	x	x	address	<AAAAAAAAAAAAAAAAAAAA>		
KELURAHAN	x	x	village office	## (dropdown box)<br>1 "KWAMKI BARU"<br>2 "KEBUN SIRIH"<br>3 "OTOMONA"<br>4 "KOPERAPOKA"<br>5 "DINGONARAMA"<br>6 "TIMIKA INDAH"<br>7 "PERINTIS"<br>8 "NAYARO"<br>9 "TIMIKA JAYA"<br>10 "NINABUA"<br>11 "HANGAITJI"<br>12 "WANAGON"<br>13 "PASAR SENTRAL"<br>14 "INAUGA"<br>15 "SEMPAN"<br>16 "KAMORO JAYA 1"<br>17 "MANDIRI JAYA"<br>18 "WONOSARI JAYA"<br>19 "KAMORO JAYA 2"<br>20 "NAWARIPI"<br>21 "MAWOKAW JAYA"<br>22 "KADUN JAYA"<br>23 "BHINTUKA"<br>24 "PIOKA KENCANA"<br>25 "MIMIKA GUNUNG"	dropdown box with village names	

				26 "UTIKINI BARU 1"<br>27 "UTUKINI BARU 2"<br>28 "UTUKINI BARU 3"<br>29 "Another Village, outside 5 Puskesmas areas"<br>30 "No village data"<br>99 "Village unknown"		
PEKERJAAN	x	x	profession	<AAAAAAAAAAAAAAAAAAAA>		
Malaria Pojok			***Malaria corner***			
KARTM		x	Has malaria card?<br>*Kartu Malaria*	# (0=no;1=yes;9=unknown)		
NKART		x	Malaria card number<br>*No. kartu malaria*	##### (#####= number)		
KPOJM		x	visiting malaria corner<br>*datang ke pojok malaria?*	# (0=no;1=yes;9=unknown)		
ATKPM		x	reason for not visiting malaria corner?<br>*alasan tidak ke pojok malaria*	## (drop down list reasons):<br>1. no malaria corner because of COVID19 - tidak ada pojok malaria karena COVID19<br>2. no malaria corner - tidak ada pojok<br>3. patient is treated outside - pasien dilayani di luar gedung<br>4. patient comes after hours - pasien di luar jam kerja<br>9. other reaons – lainnya<br>0. not applicable - tidak berlaku		
LAIPM		x	Other reason for not visiting malaria corner?<br>*alasan lain tidak ke pojok malaria?*	<AAAAAAAAAAAAAAAAAAAA>		
MINDHP1		x	took first DHP dose at malaria corner?	# (0=no;1=yes;9=unknown)	If yes 2 questions below should be 0 not filled out	

			Minum obat DHP do pojok		If no, question(s) below should be filled out	
ATMDHP		x	reason if didn't take first DHP dose<br>*alasan tidak minum DHP?*	## (drop down list reasons):<br>1. have not eaten - belum makan<br>2. cannot swallow tablet - tidak bisa telan obat<br>3. no biscuits (so first dose not offered) - tidak ada biskuit jadi tidak ditawari<br>4. no water (so first dose not offered) - tidak ada air jadi tidak ditawari<br>5. patient prefers to take it at home - mau minum obat di rumah saja<br>6. patient refused - menolak<br>7. not offered - tidak ditawari minum obat<br>8. patient already went home (their parent/others came to the malaria corner) - pasien sudah pulang<br>9. Other reason - lainnya<br>10. There was no malaria corner – tidak ada pojok<br>0. not applicable – tidak berlaku		
LAIDHP		x	Other reason didn't take first DHP dose<br>*Alasan lain tidak minum DHP*	<AAAAAAAAAAAAAAAAAAAA>		
MINPQ1		x	took first PQ dose at malaria corner?<br>*Minum obat PQ di pojok?*	# (0=no;1=yes;9=unknown)	If yes, 2 questions below should be 0 not filled out<br>If no, question(s) below should be filled out	
ATMPQ		x	reason if didn't take first PQ dose<br>*alasan tidak minum PQ?*	## (drop down list reasons):<br>1. have not eaten - belum makan<br>2. cannot swallow tablet - tidak bisa telan obat		

				3. no biscuits (so first dose not offered) - tidak ada biskuit jadi tidak ditawari<br>4. no water (so first dose not offered) - tidak ada air jadi tidak ditawari<br>5. patient prefers to take it at home - mau minum obat di rumah saja<br>6. patient refused - menolak<br>7. not offered - tidak ditawari minum obat<br>8. patient already went home (their parent/others came to the malaria corner) - pasien sudah pulang<br>9. Other reason - lainnya<br>10. There was no malaria corner – tidak ada pojok<br>0. not applicable – tidak berlaku		
LAIPQ		x	Other reason not taking first PQ dose<br>*Alasan lain tidak minum PQ?*	<AAAAAAAAAAAAAAAAAAAA>		
PENY		x	received education according to checklist?<br>*Menerima penyuluhan sesuai dengan pedoman?*	# (0=no;1=yes;9=unknown)		
Laboratorium			***Laboratory***			
LAB	x	x	lab data available	# dropdown list:<br>1 "Microscopy"<br>2 "RDT"<br>3 "Poli"<br>4 "RDT-CareStart"<br>5 "RDT-2"<br>6 "RDT-3"<br>7 "RDT-4"		

				8 "RDT-5"<br>9 Unknown		
RINGFORM	x	x	ringform	#(0=absent;1=present;9=unknown)		
GAMETOCYTE	x	x	gametocyte	#(0=absent;1=present;9=unknown)	no ring stage shouldn't get DHP?	
PF	x	x	pf	#(0=absent;1=present;9=unknown)		
PV	x	x	pv	#(0=absent;1=present;9=unknown)		
PM	x	x	pm	#(0=absent;1=present;9=unknown)		
PO	x	x	po	#(0=absent;1=present;9=unknown)		
PARASIT	x	x	Parasite name	<AAAAAAAAAAA>	Should be in accordance with species ticked in 4 questions above	
Pengobatan			***Treatment***			
DP	x	x	DP tablets	#####.## total number of DHP tablets (0 if none; 88 if given but no details; 99 if unknown)	* According to dosing table (age/) weight below per infection<br>15 tablets is max according to guidelines	
PQ	x	x	primaquine tablets	#####.## total number of PQ tablets (0 if none; 88 if given but no details; 99 if unknown)	* According to dosing table (age/) weight below per infection<br>14 tablets is max according to guidelines	
KINATAB	x	x	quinine tablets	#####.## total number of tablets (0 if none; 88 if given but no details; 99 if unknown)	Max 60 tablets?<br>Should not be given with DP or injections, unusual treatment scheme	
ARTESINJ	x	x	artesunate injection	#####.##	Can be given with DP, in combination with others very unusual treatment scheme	
ARTEMINJ	x	x	artemether injection	#####.##	Shouldn't be given in combination with others, very unusual treatment scheme	
KINAINJ	x	x	quinine injection	#####.##	Shouldn't be given in combination with others, very unusual treatment scheme	
OTHER	x	x	Other medication given	# (0=no;1=yes)		
NAMAOBAT	x	x	Name of other medication given	<AAAAAAAAAAAAAAAAAAAAA>		
COMMENTS	x	x	Comments	<AAAAAAAAAAAAAAAAAAAAA><br>free text of any comments		

Appendix 3. National Guidelines for Uncomplicated Malaria Vivax/Ovale Treatment

Days	1-3	1-14
**DRUGS**	**DHP**	**PRIMAQUINE**
**≤5 kg (0-1 months old)**	⅓	-
**>5-6 kg (2-5 months old)**	½	-
**>6-10 kg (6-12 months old)**	½	¼
**>10-17 kg (<5 years old)**	1	¼
**>17-30 kg (5-9 years old)**	1½	½
**>30-40 kg (10-14 years old)**	2	¾
**>40-60 kg (≥15 years old)**	3	1
**>60-80 kg (≥15 years old)**	4	1
**>80 kg (≥15 years old)**	5	1

Appendix 4. SHEPPI February 2022 monthly report

17 Mar 2022

SHEPPI Monthly Report: Epidemiology, Data Integrity and Patient Care Indicators

Data compiled to 26 Feb 2022

Epidemiology

In the last 30 days (between 28 January and 26 February), **2028** patients with malaria have been entered into the surveillance system across the SHEPPI clinics. Of these patients, 904 (44.6%) had falciparum malaria, 889 (43.8%) had vivax malaria, 121 (6.0%) had malariae malaria, 7 (0.3%) had ovale malaria and 106 (5.2%) had mixed species malaria. Since the inception of the SHEPPI surveillance system, 107307 cases of malaria have been recorded.

Table 1. Malaria case numbers by *Plasmodium* species and clinic during the last 30 days

	P. falciparum		*P. vivax*		*P. malariae*		*P. ovale*		Mixed		Total	
**Clinic**	N	%	N	%	N	%	N	%	N	%	N	%
PKM Timika	226	43.6	217	41.9	41	7.9	2	0.4	32	6.2	518	100
PKM Timika Jaya	145	40.8	159	44.8	25	7.0	2	0.6	24	6.8	355	100
PKM Pasar Sentral	165	40.6	218	53.7	18	4.4	0	0.0	5	1.2	406	100
PKM Wania	284	50.7	208	37.1	34	6.1	3	0.5	31	5.5	560	100
PKM Bhintuka	84	44.7	87	46.3	3	1.6	0	0.0	14	7.4	188	100
Total	904	44.6	889	43.9	121	6.0	7	0.3	106	5.2	2027	100

Table 2. Malaria patient demographics by clinic during the last 30 days

	Female		Pregnant		<1yr		1-<5yr		5-<15yr		>=15yr		Total	
**Clinic**	N	%	N	%	N	%	N	%	N	%	N	%	N	%
PKM Timika	261	50.4	15	5.7	2	0.4	61	11.8	117	22.6	338	65.3	518	100
PKM Timika Jaya	156	43.9	4	2.6	0	0.0	49	13.8	82	23.1	218	61.4	355	100
PKM Pasar Sentral	217	53.6	15	6.9	3	0.7	34	8.4	125	30.9	244	60.2	405	100
PKM Wania	261	46.5	2	0.8	9	1.6	78	13.9	134	23.9	340	60.6	561	100
PKM Bhintuka	81	43.1	1	1.2	1	0.5	24	12.8	34	18.1	129	68.6	188	100
Total	976	48.1	37	3.8	15	0.7	246	12.1	492	24.3	1269	62.6	2027	100

Fransisca et al Malaria Journal 2025
Additional File

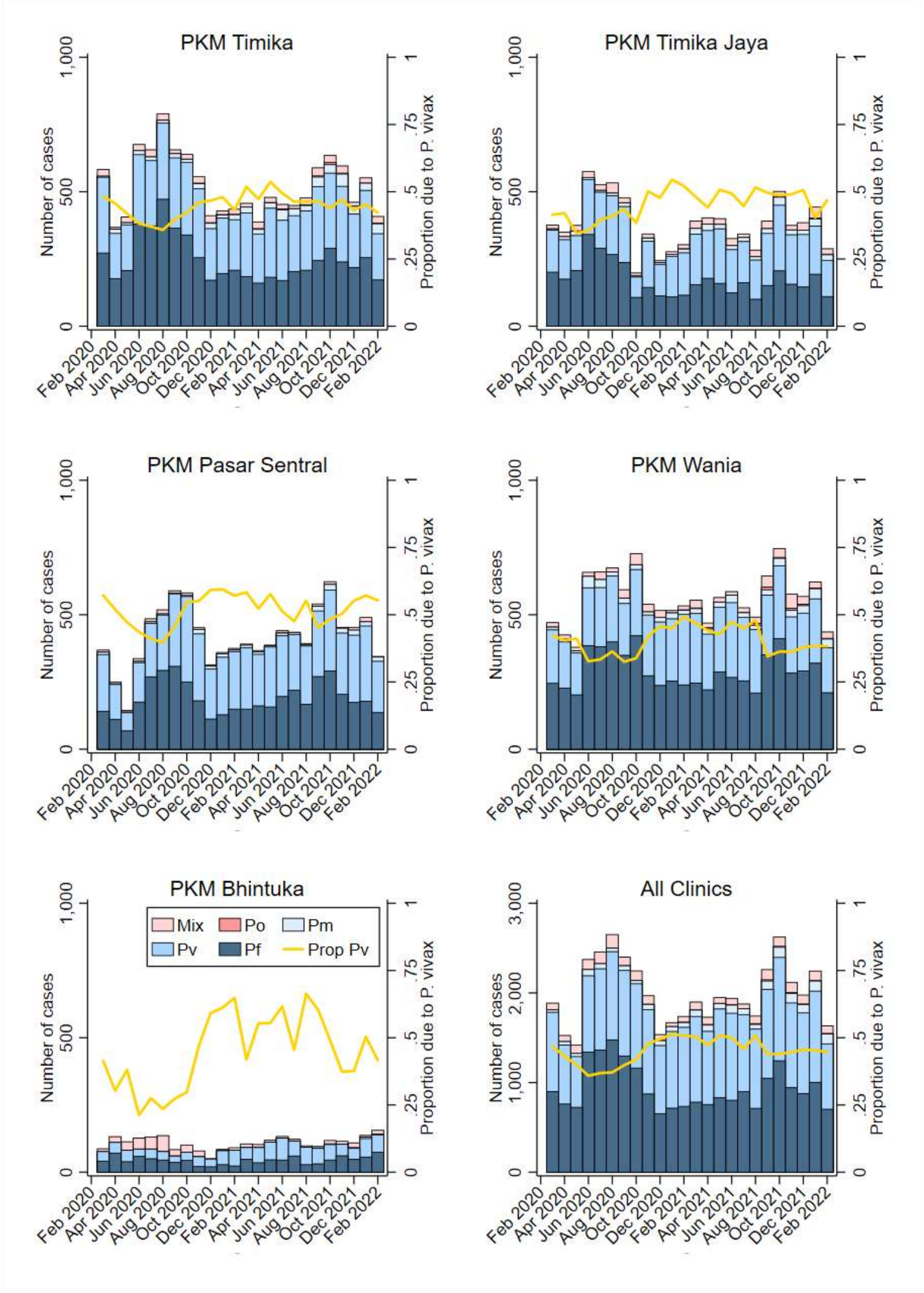

Figure 1. Number of malaria cases by *Plasmodium* species and clinic over the last 24 months

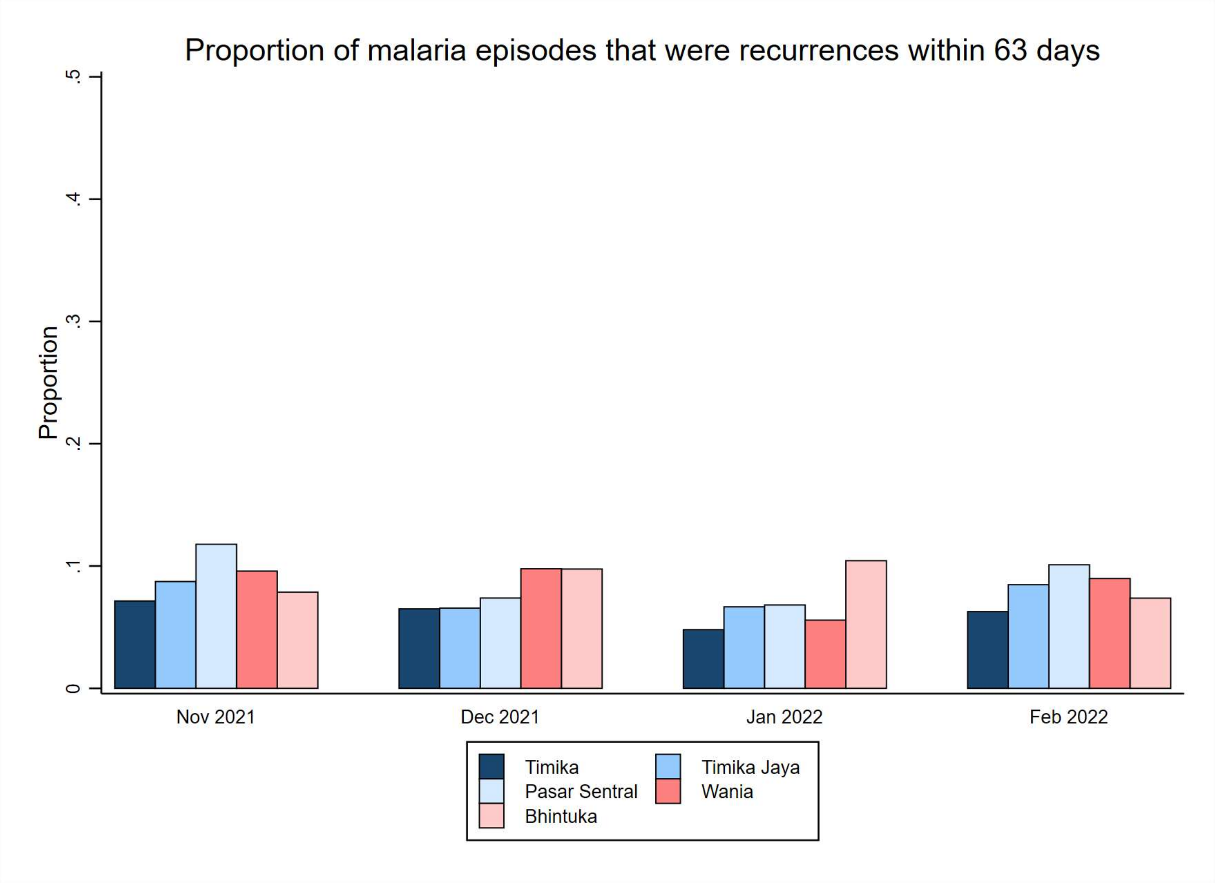

Figure 2. Proportion of malaria cases that were recurrences within 63 days

Table 3. Number and proportion of malaria cases in the last 30 days that were recurrences, by species and clinic. Limited to patients assigned a malaria card number.

	P. falciparum					*P. vivax*					Mixed species				
	Total Pf	Recurrence within 63d		Recurrence within 180d		Total Pv	Recurrence within 63d		Recurrence within 180d		Total mix	Recurrence within 63d		Recurrence within 180d	
**Clinic**		N	%	N	%		N	%	N	%		N	%	N	%
PKM Timika	157	7	4.5	29	18.5	140	7	5.0	37	26.4	23	3	13.0	8	34.8
PKM Timika Jaya	122	9	7.4	20	16.4	120	12	10.0	23	19.2	22	6	27.3	6	27.3
PKM Pasar Sentral	136	13	9.6	39	28.7	178	18	10.1	47	26.4	4	1	25.0	1	25.0
PKM Wania	227	19	8.4	54	23.8	158	12	7.6	42	26.6	11	1	9.1	5	45.5
PKM Bhintuka	72	4	5.6	8	11.1	65	5	7.7	15	23.1	9	0	0.0	0	0.0
All clinics	714	52	7.3	150	21.0	661	54	8.2	164	24.8	69	11	15.9	20	29.0

Table 4. Kaplan-Meier failure rates at day 63 for recurrence of *P. vivax* or mixed species infection by clinic and receipt of first dose of primaquine at the malaria corner. Excludes infants and pregnant women, who are ineligible for primaquine. Includes presentations over the last 6 months.

**Clinic**	Pq at mal cor	N at risk	Risk of failure (%)	Lower bound (%)	Upper bound (%)
PKM Timika	No	225	5.98	3.75	9.48
	Yes	506	4.56	3.15	6.59
PKM Timika Jaya	No	268	9.40	6.72	13.08
	Yes	172	6.55	3.90	10.89
PKM Pasar Sentral	No	32	16.20	8.49	29.68
	Yes	708	5.54	4.19	7.30
PKM Wania	No	229	11.61	8.48	15.79
	Yes	476	3.49	2.24	5.44
PKM Bhintuka	No	30	6.06	1.55	22.12
	Yes	131	10.93	6.99	16.88
All clinics	No	779	9.39	7.74	11.37
	Yes	1992	5.29	4.46	6.28

Table 5. Kaplan-Meier failure rates at day 63 for recurrence of *P. falciparum* infection by clinic and receipt of first dose of dihydroartemisinin-piperaquine at the malaria corner. Includes presentations over the last 6 months.

**Clinic**	DHP at mal cor	N at risk	Risk of failure (%)	Lower bound (%)	Upper bound (%)
PKM Timika	No	171	2.45	1.01	5.87
	Yes	525	3.40	2.23	5.18
PKM Timika Jaya	No	208	4.91	2.92	8.19
	Yes	147	3.21	1.43	7.10
PKM Pasar Sentral	No	11	12.34	2.84	45.20
	Yes	730	3.90	2.79	5.45
PKM Wania	No	218	5.95	3.76	9.34
	Yes	839	2.76	1.89	4.03
PKM Bhintuka	No	18	6.18	1.56	22.78
	Yes	111	4.23	1.89	9.34
All clinics	No	615	4.82	3.55	6.52
	Yes	2351	3.36	2.75	4.11

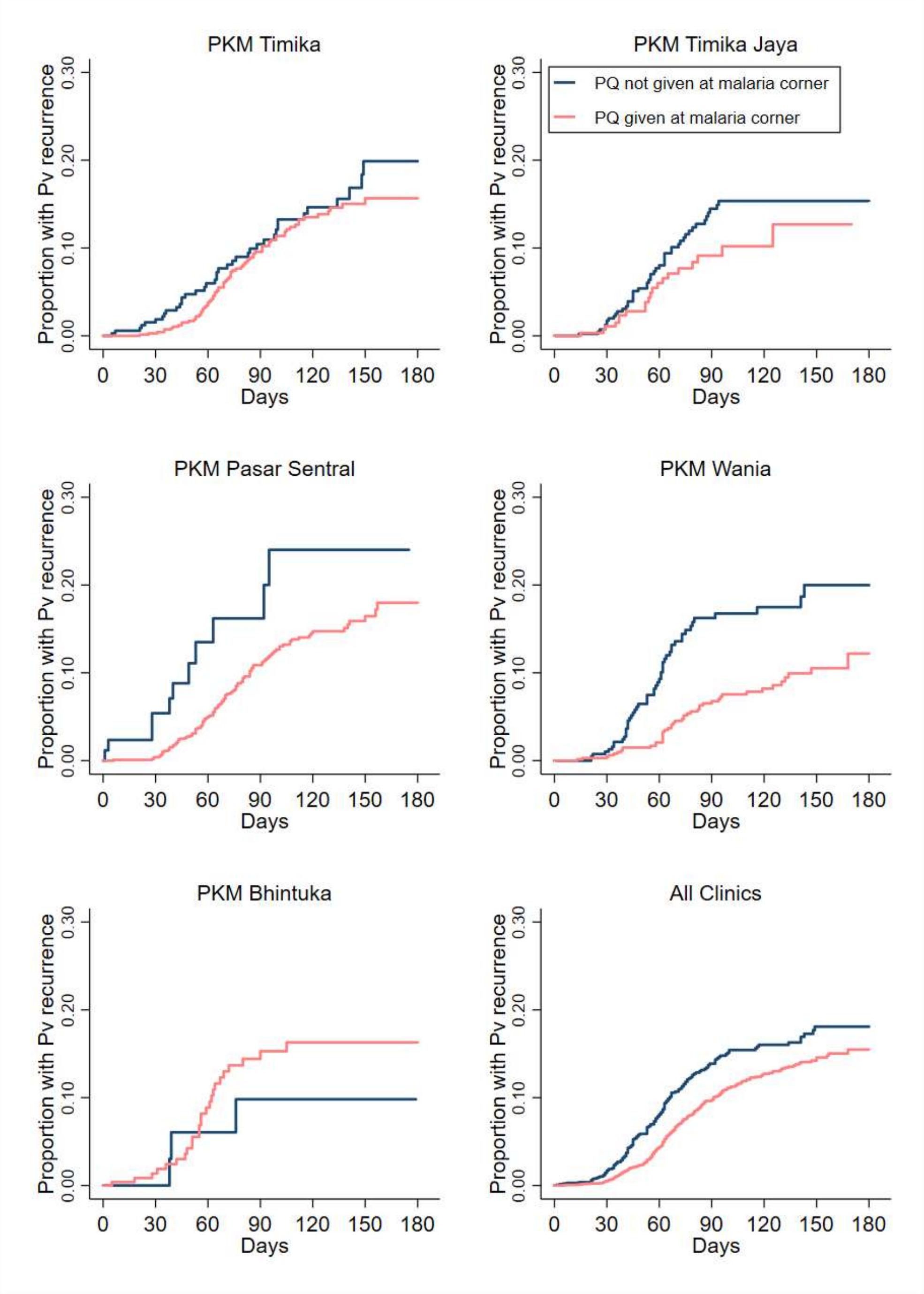

Figure 3. Kaplan-Meier figures showing cumulative proportion of patients with *Plasmodium vivax* or mixed species infection who have recurrence of *P. vivax* or mixed species infection within 180 days according to whether or not they received their first dose of primaquine at the malaria corner (NB: excludes infants and pregnant women, who are ineligible for primaquine). Includes presentations over the last 6 months.

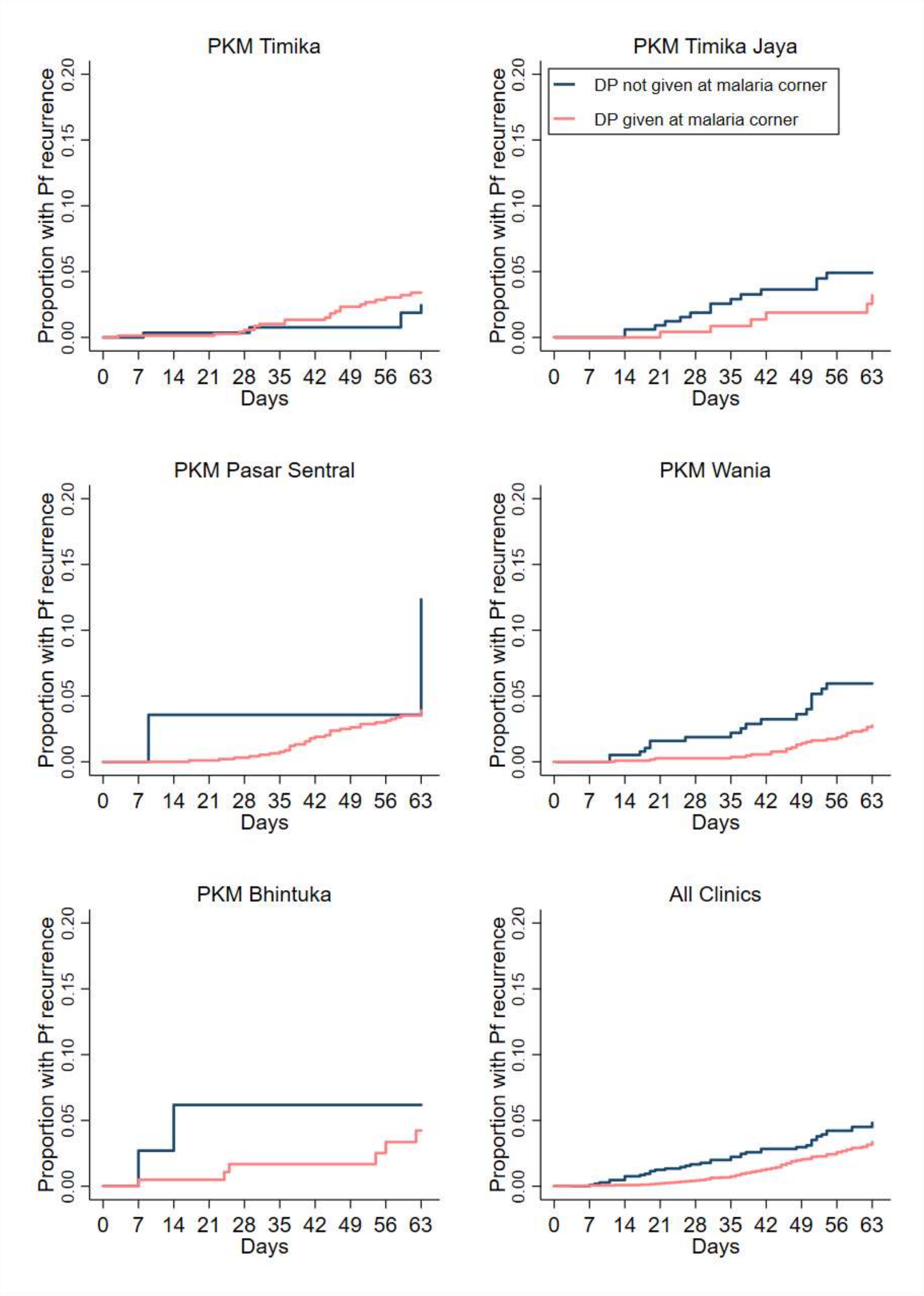

Figure 4. Kaplan-Meier figures showing cumulative proportion of patients with *Plasmodium falciparum* monoinfection who have *P. falciparum* monoinfection recurrence within 63 days according to whether or not they received their first dose of dihydroartemisinin-piperaquine at the malaria corner. Includes presentations over the last 6 months.

Data Integrity

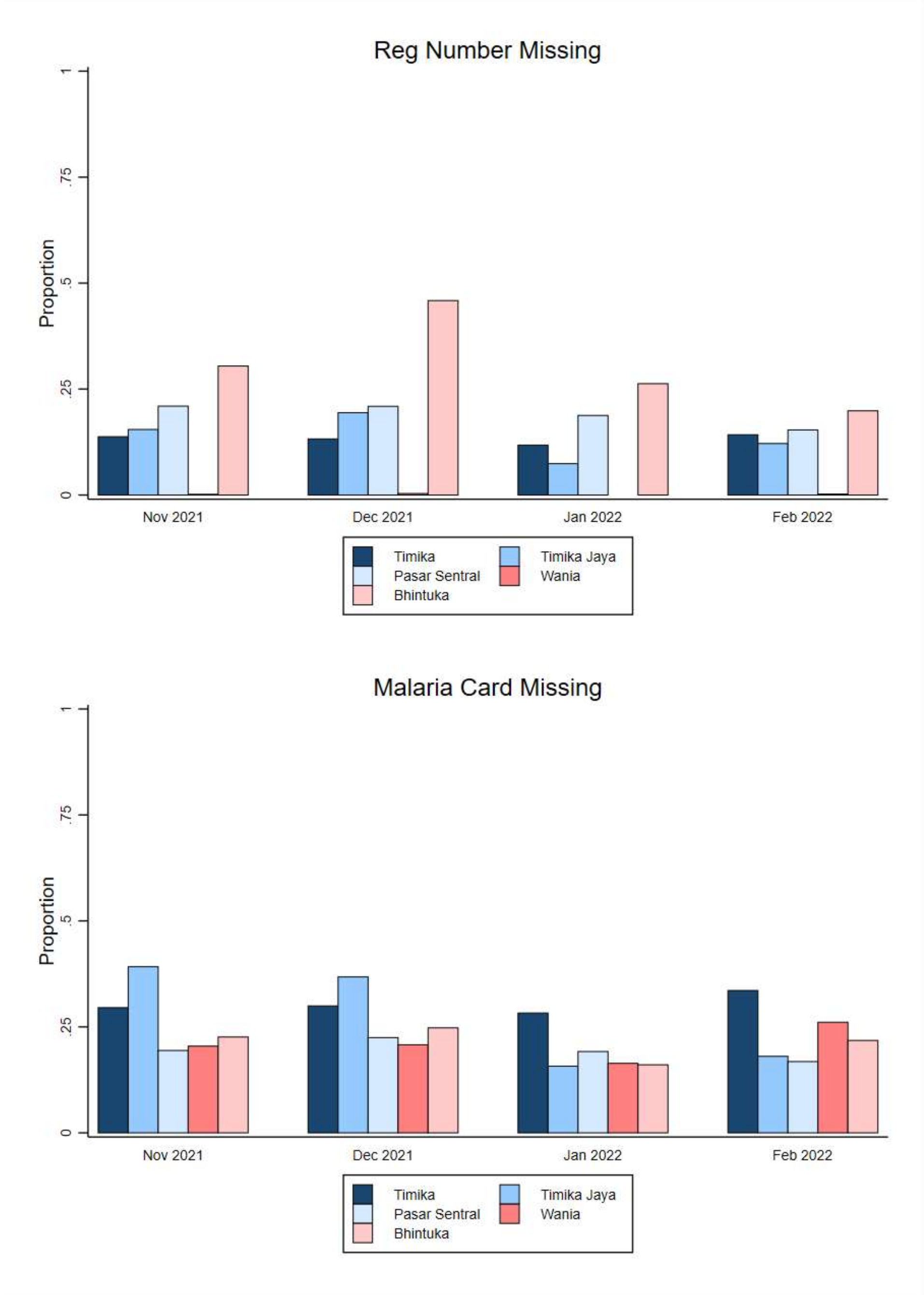

Figure 5. Proportion of patients with registration number or malaria card missing by clinic over the last 4 months

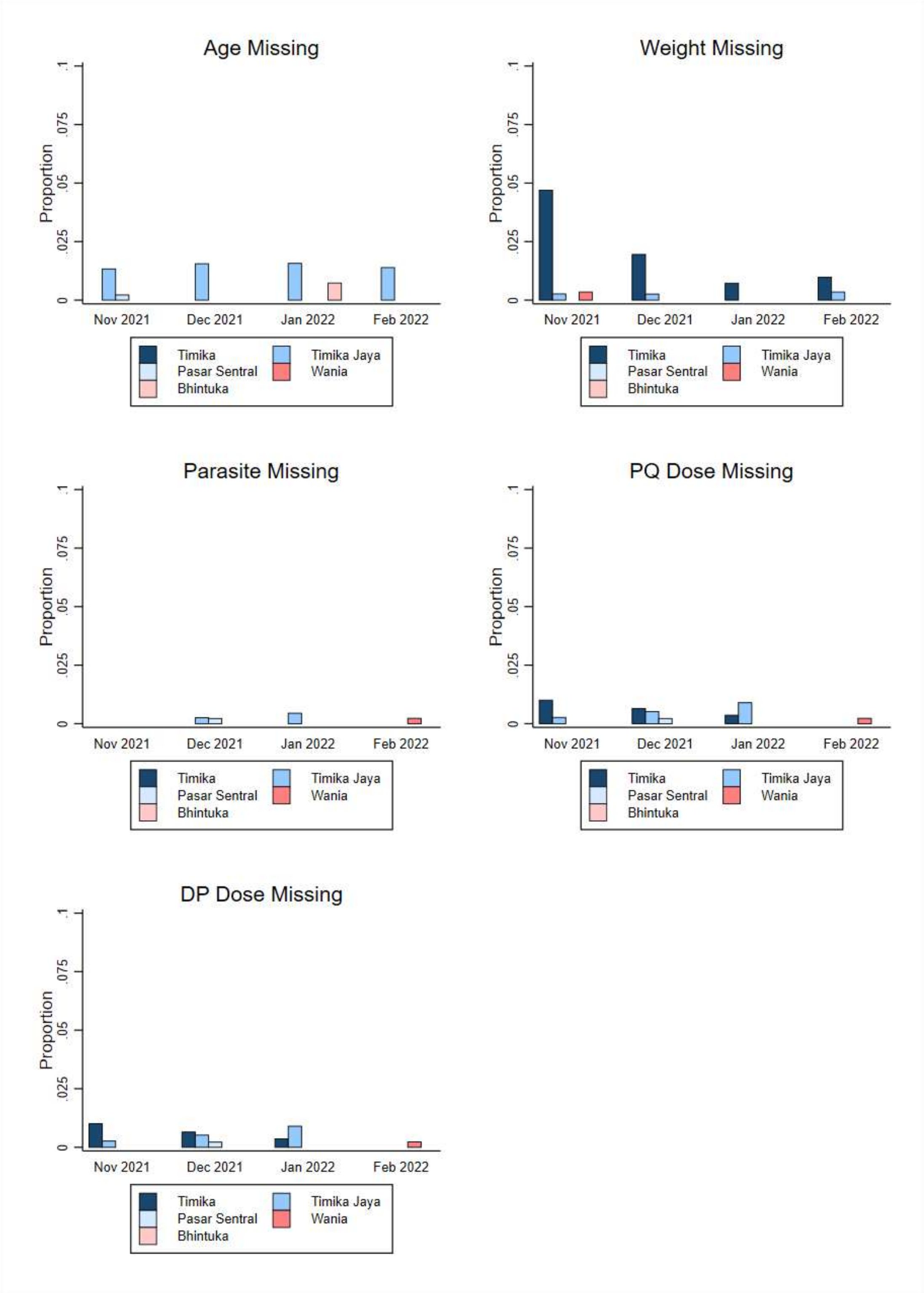

Figure 6. Proportion of patients with age, weight, parasite, primaquine dose or dihydroartemisinin-piperaquine dose missing by clinic over the last 4 months

Patient Care Indicators

Table 6. Number of pregnant women given primaquine in the last 30 days by clinic

	Pregnant women given PQ		
**Clinic**	N	%	Total pregnant women
PKM Timika	0	0	15
PKM Timika Jaya	0	0	4
PKM Pasar Sentral	1	6	16
PKM Wania	0	0	2
PKM Bhintuka	0	0	1

Table 7. Number of infants <5kg (or <6 months if weight not available) given primaquine in the last 30 days by clinic

	Infants given PQ		
**Clinic**	N	%	Total infants
PKM Timika	0	.	0
PKM Timika Jaya	0	0	1
PKM Pasar Sentral	0	.	0
PKM Wania	0	.	0
PKM Bhintuka	0	0	1

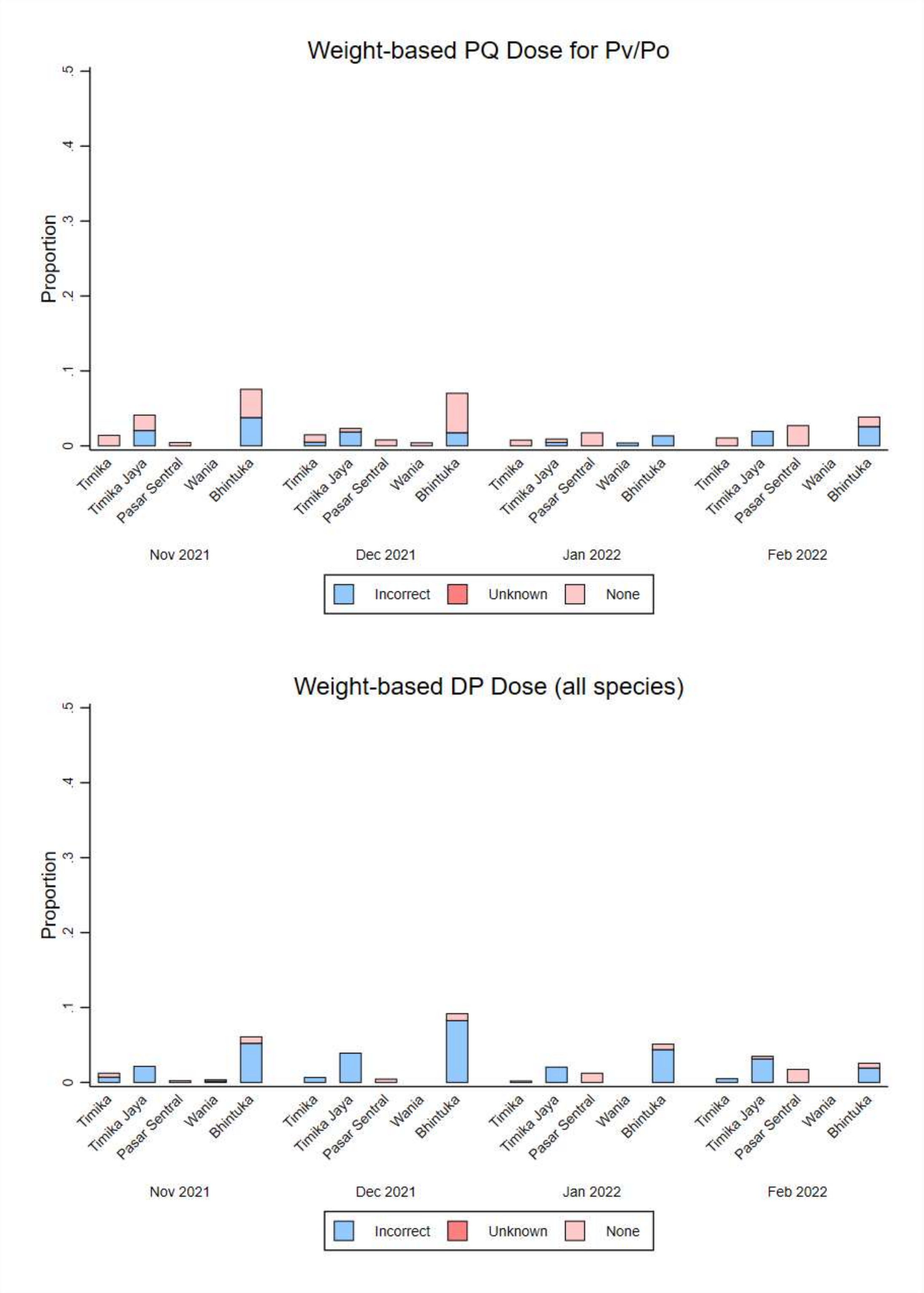

Figure 7. Appropriateness of weight-based dosing of primaquine and dihydroartemisinin-piperaquine by clinic over the last 4 months (remainder prescribed correctly)

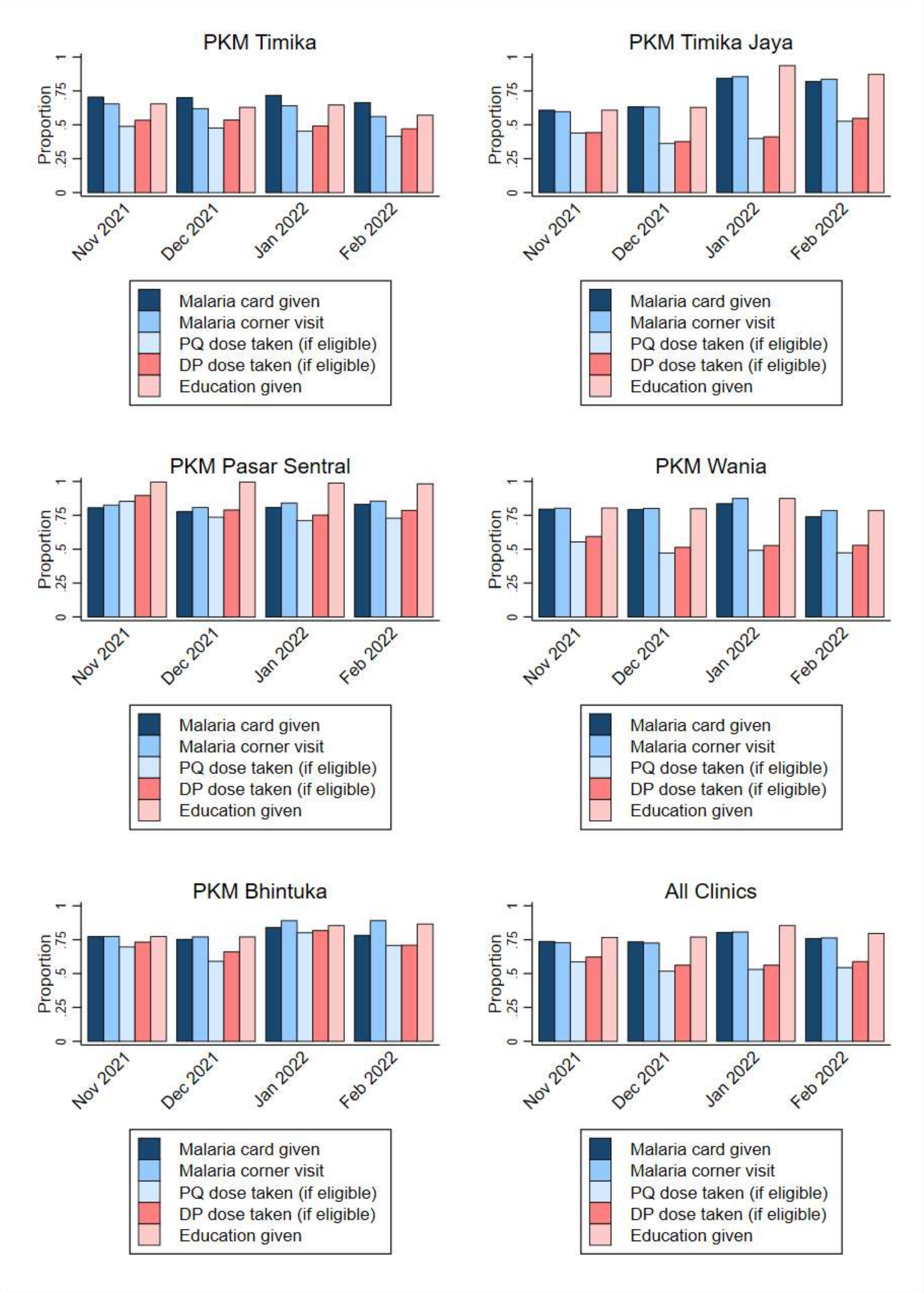

Figure 8. Cascade of malaria care by clinic over the last 4 months

Appendix 5. Topics of Continuous Quality Improvement (CQI) workshops

CQI	Year	Month	Attendees	Issues Raise
1	2019	May	35	• Data completeness and accuracy
2	2019	August	18	• The concept of enhanced patient engagement through attendance at the 'malaria corners' and community health workers (CHWs)<br>• Collection of quality data
3	2020	March		• Patient education<br>• Collection of quality data
4.	2020	August		• Antimalarial prescription according to the national guideline<br>• Treatment adherence<br>• Collection of quality data
5	2020	Sept		• Detecting recurrences<br>• Collection of quality data
6	2021	March	28	• Collection of quality data<br>• Qualitative analysis findings on the role of malaria CHWs and clinic program officers
7	2021	July	25	• Regency Health Office Decree on the Role of Malaria CHWs in Mimika<br>• Allocation of CHWs' work zones, malaria patient referral and household screening<br>• Collection of quality data
8	2021	November	25	• First dose supervision<br>• Patient phone follow up<br>• Challenges in patient referral to CHWs, treatment monitoring and patient's household screening by CHWs
9	2022	March	34	• Malaria recurrence<br>• Patients referral to CHWs
10	2023	March	28	• Cascade of care and effective communication at malaria corner<br>• Unique identifier in detecting recurrence
11	2023	September	39	• Malaria corner operational<br>• Patients referral to CHWs

Appendix 6. SHEPPI Statistical Analysis Plan

SHEPPI: Statistical Analysis Plan

Paper 1

Table of Contents

Table of Contents **24**
1. **Introduction and Rationale** **25**
2. **Outline of Statistical Analysis** **25**
   2.1 Specific objectives of the study 25
   2.2 Endpoints 25
   2.3 Exposures of Interest 26
   2.4 Definitions 26
3. **Summary of statistical analyses** **27**
   3.1 Description and baseline characteristics 27
   3.2 Data Quality: 27
   3.3 Quality of Clinical Management 28
4 **Tools** **29**

1. Introduction and Rationale

Mimika Regency, located in the southern part of Papua, has the highest burden of malaria in Indonesia, with Annual Parasite Incidence more than 400 times higher than the national average. Cases are concentrated mainly in Timika, the capital city. A good understanding of the epidemiology of malaria and current treatment practices is required to optimize effective control and elimination strategies. District-specific malaria surveillance and targeted response is pivotal for effective malaria control in Mimika.

The national electronic malaria surveillance system (eSISMAL) is used by public government hospitals and clinics. Its completeness and timeliness are often an issue for health facilities in the rural part of high-endemic areas with limited computer and internet access, such as in Mimika. Health clinics in Mimika typically have a poor medical record system. In addition, patients often forget to bring their medical cards, and healthcare providers often fail to address this by retrieving medical records. Robust measures of the true burden of disease require quality surveillance.

It is also hard to estimate the risk of recurrence without a unique patient identification system to identify representations reliably. Incomplete surveillance data undermines the interpretation of case trends and the associated impact of malaria control practices and interventions. Robust malariometric surveillance is critical for optimizing healthcare, which can be improved with simple and standardized data collection and timely feedback to healthcare staff.

2. Outline of Statistical Analysis

2.1 Specific objectives of the study

- Describe the epidemiology and current management of malaria in Timika, Papua.
- Assess the impact of enhanced malariometric surveillance and data feedback on the quality of malaria care in primary healthcare centers in Timika, Papua.

2.2 Endpoints

All endpoints will be calculated using data entered into the database for the study period of January 2019 to December 2023 and will be presented monthly unless stated otherwise.
The database used was EpiData (until November 2022), and it continued with REDCap.

2.2.1. Primary Endpoint:

- Number of patients diagnosed with malaria stratified by species

2.2.2. Secondary Malariometric Endpoints:

- Slide positivity rates for each Plasmodium species
- Proportion of patient presentations identified as recurrent visits within 6 months

2.2.3. Secondary Endpoints – Data Quality:

- Proportion of patients with missing register numbers
- Proportion of patients with missing age data
- Proportion of patients with missing weight data

- Proportion of patients with missing gender data
- Proportion of female patients with missing pregnancy status
- Proportion of patients with missing parasite data
- Proportion of patients assigned a malaria card number
- Proportion of patients with missing data to calculate DHP prescription accuracy
- Proportion of patients with missing data to calculate PQ prescription accuracy

2.2.4. Secondary Endpoints – Quality Management:

- Proportion of patients with malaria due to any Plasmodium species correctly prescribed the recommended age/weight-based dose of DHP according to the national guidelines
- Proportion of patients with *P. vivax* or *P. ovale* malaria correctly prescribed the recommended age/weight-based dose of PQ according to the national guidelines
- Proportion of infants < 6 months of age or <6 kg inappropriately prescribed PQ
- Proportion of pregnant women inappropriately prescribed PQ

2.3 Exposures of Interest

2.3.1. Factors affecting malaria epidemiology:

Site **:** clinic, drug stock-out period
Patient **:** age, sex, pregnancy

2.3.2. Factors affecting data quality

Site : clinic, staffing
Interventions : CQI workshops

2.4 Definitions

2.4.1. Age Groups

Children will be considered as any patient aged ≤15 years.
Childhood will be stratified into patients <1 years, 1-5 years, and those >5-15 years.

2.4.2. Prescribing Accuracy

Prescribing accuracy will be measured based on the number of tablets directed by the national guideline on malaria treatment (Buku Saku Penatalaksanaan Kasus Malaria, 2020).
ACT Underdosing : Patients receiving fewer DHP tablets than directed in guideline
ACT Overdosing : Patients receiving more DHP tablets than directed in guideline
PQ Underdosing : Patients receiving fewer PQ tablets than directed in guideline
PQ Overdosing : Patients receiving more PQ tablets than directed in guideline

3. Summary of statistical analyses

3.1 Description and baseline characteristics

Descriptive summary statistics of baseline diagnostic methods, treatment algorithms (including dosing and exclusions), second-line regimens used during stock outages, and data collection processes will be presented.

Slide Positivity Rate (SPR) between 2019 and 2023 will be derived from laboratory records at each clinic and calculated as the number of positive malaria slides divided by the total number of blood films examined. SPR will be stratified by species, and presented by month for the observational period. RDT results will not be considered.

The absolute number of malaria cases stratified by species will be presented over time (monthly). Malaria cases will be temporally related to key events such as stock outs and the COVID-19 pandemic. The proportion of malaria patient attending any of the study clinics will be presented per year, stratified by species, clinic, and age.

A summary of relevant baseline patient characteristics will be presented for all malaria patients. Variables presented will include parasite species, age group, weight by age groups, sex, pregnancy, and lactating status. Depending on data distribution, continuous data will be presented as mean and 95% confidence interval (95%CI) or median and inter-quartile range (IQR).

Table: Microscopy Slide Positive Rate for each Plasmodium species, 2019-2023
Figure: Microscopy Slide Positive Rate (SPR) for each Plasmodium species and clinic by month, 2019-2023
Table: Baseline Characteristic by clinic, 2019-2023
Figure: Malaria cases per month by Plasmodium species, 2019-2023
Figure: The proportion of malaria patients attending clinics each year, stratified by Plasmodium species, 2019-2023
Figure: The proportion of malaria patients, stratified by Plasmodium species and age group, 2019-2023

3.2 Data Quality:

The proportions of missing Data Quality Endpoints (Section 2.2) will be presented for each metric. Variables likely to determine data quality will be explored including clinic and the number of staff in malaria corner.

The proportion of patients with available data to determine DHP and PQ dose will be presently. The impact of CQI will be assessed by comparing the proportion of missing data in the 5 months before and 5 months after the first and second CQI.

In the first 5 months, data integrity to calculate DHP and PQ prescription accuracy will be tabulated for different patients characteristics (clinic, age group, and gender) to identify populations at greatest risk of missing data.
Figure: Proportion of missing data needed to calculate DHP and PQ Prescription Accuracy, by month
Figure: Data Integrity, 2019-2023

3.3 Quality of Clinical Management

3.3.1 Primaquine Administration

The proportion and absolute numbers of patients diagnosed with *P. vivax* or *P. ovale* malaria who were and were not prescribed PQ will be presented. The prescribing accuracy will be presented in terms of overdosing (too many tablets) and underdosing (too few tablets) according to the recommended age/weight-based PQ in the national antimalarial guidelines. These data will be graphed over time (monthly) and temporally related to key events such as CQI and drug stock outs.

For all patients with available data, the mg/kg total dose of PQ administered will be calculated and presented as a histogram. Reference lines will mark 3.5 mg/kg to indicate the low dose used in Indonesia and at 7 mg/kg to indicate the high dose that should be more optimum for high relapse periodicity area.

Pooled quarterly prescribing accuracy will also be presented. Accuracy will be compared from first 5 months of 2019 (pre CQI), second 5 months of 2019 (post CQI) and last 5 months of 2023. For the first 5 months of 2019, the risk of over and under dosing will be presented in table for different patients characteristics (clinic, age group, and gender) to determine populations at greatest risk of incorrect dosing.

Frequency of inappropriate treatment will be graphed over time (monthly) for both pregnant women and infants.

3.3.2 DHP Administration

The proportion and absolute numbers of patient diagnosed with any malaria who were and were prescribed DHP will be presented. The prescribing accuracy will be presentred in terms of overdosing (too many tablets) and underdosing (too few tablets) according to the recommended age/weight-based DHP in the national antimalarial guidelines. These data will be graphed over time (monthly) and temporally related to key events such as CQI and drug stock outs.

For all patients with available data, the mg/kg daily dose of DHA administered will be calculated and presented as a histogram, with reference line at 2 and 4 mg/kg to indicate the range of optimal dose. The same approach will be used for piperaquine, with reference line at 16 and 32 mg/kg.

Pooled quarterly prescribing accuracy will also be presented. Accuracy will be compared for the first 5months of 2019 (pre CQI), the second 5 months of 2019 (post CQI) and last 5 months of 2023. For the first 5 months of 2019, the risk of over and under dosing will be presented in table for different patients characteristics (clinic, age group, and gender) to determine populations at greatest risk of incorrect dosing.

Figure: Dosing Distribution for DHP and PQ, 2019-2023
Figure: Proportion of no dose for DHP and PQ by month
Figure: Dosing distribution Q1 2019 versus Q4 2023 for DHP and PQ
Figure: Histogram of mg/kg dosing for DHA, Piperaquine, and Primaquine

Table: Data availability and Dosing Accuracy of DHP and PQ Among Malaria Patients
Table: Data availability and Dosing Accuracy of DHP and PQ Stratified by Clinic, Age Group, and Sex
Figure: Frequency of inappropriate treatment in pregnant women by month
Figure: Frequency of inappropriate treatment in infants by month

4 Tools

All statistical analyses will be carried out using Stata (Stata Corp, College Station, Texas). However, when equivalent statistical methods are applied in a different statistical software package such as R, amendment of this SAP is not required.
